# Small RNA pyrosequencing in the protozoan parasite *Entamoeba histolytica* reveals strain-specific small RNAs that target virulence genes

**DOI:** 10.1186/1471-2164-14-53

**Published:** 2013-01-25

**Authors:** Hanbang Zhang, Gretchen M Ehrenkaufer, Neil Hall, Upinder Singh

**Affiliations:** 1 Division of Infectious Diseases, Department of Internal Medicine, Stanford University School of Medicine, Stanford, California, 94305-5107, USA; 2 School of Biological Sciences, Biosciences Building, University of Liverpool, Crown Street, Liverpool, L69 7ZB, UK; 3 Department of Microbiology and Immunology, Stanford University School of Medicine, Stanford, California, 94305-5107, USA; 4 Department of Medicine, Division of Infectious Diseases, S-143 Grant Building, 300 Pasteur Drive, Stanford, CA, 94305, USA

**Keywords:** 5^′^-Polyphosphate small RNA, Argonaute, High-throughput pyrosequencing, Parasite

## Abstract

**Background:**

Small RNA mediated gene silencing is a well-conserved regulatory pathway. In the parasite *Entamoeba histolytica* an endogenous RNAi pathway exists, however, the depth and diversity of the small RNA population remains unknown.

**Results:**

To characterize the small RNA population that associates with *E. histolytica* Argonaute-2 (EhAGO2-2), we immunoprecipitated small RNAs that associate with it and performed one full pyrosequencing run. Data analysis revealed new features of the 27nt small RNAs including the 5^′^-G predominance, distinct small RNA distribution patterns on protein coding genes, small RNAs mapping to both introns and exon-exon junctions, and small RNA targeted genes that are clustered particularly in sections of genome duplication. Characterization of genomic loci to which both sense and antisense small RNAs mapped showed that both sets of small RNAs have 5^′^-polyphosphate termini; strand-specific RT-PCR detected transcripts in both directions at these loci suggesting that both transcripts may serve as template for small RNA generation. In order to determine whether small RNA abundance patterns account for strain-specific gene expression profiles of *E. histolytica* virulent and non-virulent strains, we sequenced small RNAs from a non-virulent strain and found that small RNAs mapped to genes in a manner consistent with their regulation of strain-specific virulence genes.

**Conclusions:**

We provided a full spectrum analysis for *E. histolytica* AGO2-2 associated 27nt small RNAs. Additionally, comparative analysis of small RNA populations from virulent and non-virulent amebic strains indicates that small RNA populations may regulate virulence genes.

## Background

RNA interference (RNAi) is a well-conserved gene regulatory pathway found in most eukaryotes [1,2]. Many important biological functions are controlled by RNAi such as developmental regulation [3], genome protection against viruses and transposons [4], and DNA elimination [5]. Small RNA (sRNA) molecules, usually 20 – 30nt, are the key elements for RNAi. Guided by their associated protein complexes, they base-pair to the targeted transcripts or genomic loci to trigger gene silencing at either the transcriptional or post-transcriptional level [6]. In recent years, high throughput sequencing has facilitated the identification of diverse species of small RNAs in different organisms [7].

Several protozoan parasites such as *Trypanosoma brucei, Toxoplasma gondii*, *Giardia lamblia*, *Trichomonas vaginalis*, and *Entamoeba histolytica* contain key genes of the RNAi pathway in their genomes [8,9]. The functions of RNAi in parasite biology include retrotransposon control in *T. brucei*[10], gene regulation in *E. histolytica*[11,12], and control of antigenic variation in *G. lamblia*[13,14]. In *T. gondii* and *T. vaginalis* the studies have been limited to endogenous small RNA sequencing with no functional studies yet reported [15,16].

*E. histolytica* causes dysentery and liver abscesses in humans and affects 500 million people worldwide [17]. The study of this important human parasite has been hampered by lack of standard molecular genetic tools due to the polyploid nature of the *E. histolytica* genome [18]. Recently, several RNAi-based gene knockdown approaches -- dsRNA/siRNA [19,20], short-hairpin RNA [21] and a transcriptional gene silencing approach in the G3 parasite strain [22] -- have been established in this organism. We have shown that *E. histolytica* has a 27nt small RNA population, which has 5^′^-polyphosphate (5^′^-polyP) and 3^′^-OH termini and associates with EhAGO2-2 [11]. Additionally, we have demonstrated that gene silencing in the *E. histolytica* G3 strain is mediated through a siRNA pathway [12].

The *E. histolytica* genome encodes three Argonaute proteins (EHI_125650, EHI_186850, and EHI_177170) of which EhAGO2-2 (EHI_125650) is highly expressed and associates with 27nt small RNAs [11]. In this report, we immunoprecipitated small RNAs bound to EhAGO2-2 and sequenced them using a high throughput pyrosequencing approach, which generated over 360,000 small RNA reads. Analysis of these endogenous small RNAs revealed that their peak-length is 27nt and that there was a strong G bias at the 5^′^-nucleotide. Genome analysis showed that small RNAs largely mapped to annotated protein coding genes (overall mapping to ~4% of protein coding genes in the genome), which can be categorized into three groups: (I) genes with only antisense small RNAs; (II) genes with both antisense and sense small RNAs; and (III) genes with only sense small RNAs. Biochemical analysis revealed that both sense and antisense small RNAs (that map to group I or II genes) have 5^′^-polyP termini. Strand specific RT-PCR showed both sense and antisense transcripts can be detected for group II gene loci, suggesting that bi-directional transcripts are available to serve as templates for small RNA generation. Identification of small RNAs that map to introns as well as exon-exon junctions indicated that *E. histolytica* RNA-dependent RNA polymerase (RdRP) could use both nascent and mature transcript as a template for generating small RNAs. Whole genome microarray data indicated that most protein coding genes with large numbers of antisense small RNAs are not expressed, indicating that antisense small RNAs likely play a role in gene silencing. We further sequenced a size-fractionated small RNA library from the non-virulent *E. histolytica* Rahman strain and identified strain-specific patterns of antisense small RNAs mapping to a virulence gene in a manner consistent with its regulation by small RNAs. In summary, we have characterized the endogenous small RNA populations from a virulent and non-virulent strain of *E. histolytica* using a high-throughput small RNA pyrosequencing approach. Our analysis indicates that the small RNA pathway likely regulates expression of strain-specific genes in this parasite system, including some virulence determinants.

## Results

### High-throughput pyrophosphate sequencing of EhAGO2-2-bound small RNAs

A library of EhAGO2-2 associated small RNAs was generated from RNA obtained by immunoprecipitation (IP) with anti-Myc antibody in *E. histolytica* HM-1:IMSS cells stably expressing Myc-tagged EhAGO2-2. We have previously shown that EhAGO2-2 specifically associates with 27nt small RNAs, which have 5^′^-polyP and 3^′^-OH termini [11]. Thus, the small RNA library was generated using a 5^′^-P independent cloning approach (Table 1). 454 Genome Sequencer process-specific A and B adaptors were incorporated into the cDNA small RNA library by PCR and a full sequencing run was performed generating a total of 362,445 sequences. Using the small RNA sequence analysis workflow (Additional file 1: Figure S1), we removed adaptor and linker sequences and limited the size to 15-40nt. The resulting output contained 340,280 sequences that represented 209,513 unique sequence reads. About 75.8% (158,904) of the unique sequences were cloned only once indicating that the current sequencing was far from saturating (Table 1).

**Table 1:** **The algorithm for small RNA processing for the EhAGO2-2 IP small RNA library from *E. histolytica *HM-1:IMSS**

**Procedures**	**Output**
5^′^-P independent cloning (5^′^CIP+PNK method)	HM-1:IMSS AGO2-2 IP library
Total reads by Pyrosequencing	362,445 reads
Primer trimming, size-limiting (15–40nt)	340,280 reads
Unique sequences (percentage)	209,513 unique sequences (158,904 cloned only once; 75.8%) (50,609 cloned more than once; 24.2%)
Scan for tRNA, rRNA	tRNA: 1,640 sequences rRNA: 3,456 sequences
Scan for SINE/LINE, EhERE elements	8,073 sequences
Map to *E. histolytica* genome	**140,943 sequences**
Map to *E. histolytica* predicted ORFs	100,190 sequences

One pyrophosphate sequencing run was performed. Unix tools were used to remove adaptor and linker sequences. The Bowtie alignment tool was used to scan for structural RNAs, repetitive elements and for the final genome mapping. The numbers of reads that map to the genome sequence at each step are listed.

**Figure 1:**
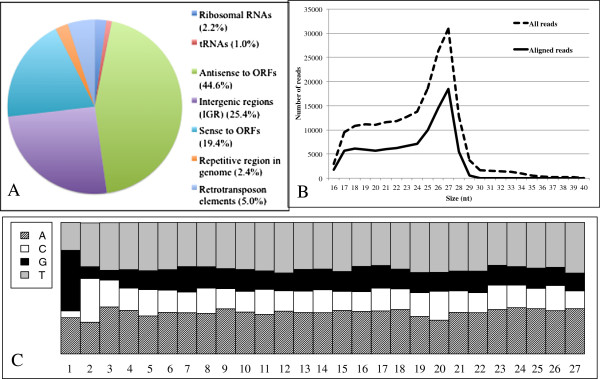
**Characterization of endogenous small RNAs that bind EhAGO2-2 in *E. histolytica *HM-1:IMSS trophozoites. **(**A**) The composition of small RNAs from the EhAGO2-2 IP small RNA library that match the *E. histolytica* genome annotation in the HM-1:IMSS strain. (**B**) Small RNA length distribution for all reads (dashed black line) and all reads remaining after filtering out those that originate from structural RNAs and repetitive elements reads (solid black line) is shown. (**C**) Nucleotide frequency at each position for the aligned 27nt reads reveals a 5^′^-G predominance

We used the Bowtie alignment tool [23] to filter out reads that mapped to structural RNAs: tRNA (1,640 reads), rRNA (3,456 reads) and repetitive elements (EhSINEs, EhLINEs and EhERE elements) (8,073 reads). The remaining dataset (196,343 reads) was mapped to the *E. histolytica* HM-1:IMSS genomic sequence. A total of 140,943 reads mapped to the genome (with perfect match or one nucleotide mismatch) with 100,190 reads mapping to open reading frames and 85,000 reads mapping to a single gene locus (Additional file 1: Figure S2). This dataset targets ~4% of annotated protein coding genes in the *E. histolytica* genome (using ≥ 50 small RNAs mapping to each gene as a cutoff). The genome mapping revealed some striking features. First, many small RNA reads mapped antisense to annotated protein coding genes (44.6%); small RNAs that mapped to intergenic regions (25.4%) and sense to protein coding genes (19.4%) were less common (Figure 1A). Second, small RNAs mapping to SINE/LINE retrotransposon elements and other potential repetitive regions in the genome only accounted for 5% and 2.4% of the reads respectively. This demonstrates that EhAGO2-2 is not primarily associated with small RNAs derived from transposons, in contrast to the single *T. brucei* Argonaute protein, which has been shown to associate with small RNAs that map primarily to transposons and are thought to control their expression [10,24,25]. Third, the mapping of small RNA to the genome indicated that small RNA reads tend to be derived from a small number of genomic locations or “hot spots”. When the genome was scanned using a 500 bp window, the majority of small RNAs arose from 1.9% of total windows in genome. This suggests that the small RNAs could associate with certain genomic features such as repeat regions, centromeric or telomeric regions, which have been shown to be a source of small RNAs in other systems [26,27]. In summary, our pyrosequencing data indicated that EhAGO2-2 associates with an abundant endogenous small RNA population, which is largely derived from the predicted protein coding genes in *E. histolytica.*

### Small RNAs are largely 27nt size with a striking 5^′^-G bias

We analyzed the size distribution and nucleotide composition of small RNAs and found that all reads (excluding reads from tRNAs, rRNAs and repetitive elements) peaked sharply at 27nt (Figure 1B), consistent with the size of small RNAs previously noted to associate with EhAGO2-2 [11]. When nucleotide frequency was plotted at each position for the 27nt population we noticed a striking 5^′^-G bias (Figure 1C). The nucleotide frequency for the 26nt and 28nt small RNAs also shows a 5^′^-G propensity, but this was not the case for 17nt small RNAs indicating that the smaller sequences are likely degradation products (Additional file 1: Figure S3). Given that the *E. histolytica* genome is very AT-rich (approximately 75% AT) [28], the 5^′^-G bias in small RNAs is remarkable when compared to all the remaining plotted positions for small RNAs. A 5^′^-G bias in small RNAs has been reported in two other organisms, i.e. *C. elegans* and *Ascaris*, where 22G populations with 5^′^-polyP termini are defined as secondary siRNAs and thought to be generated by RdRP [29,30]. Thus, we reason that the 5^′^-G biased 27nt small RNAs are likely RdRP-related and function as silencing siRNAs in *E. histolytica*.

### Small RNA distribution patterns in the genome

The *E. histolytica* genome was first published in 2005 [31] and a second version including new assemblies and reannotation was released in 2010 [32]. The current genome assembly is still in scaffold stage, which contains 1,496 supercontigs and 8,201 genes. To gain an overview of small RNA distribution in the genome, we mapped small RNAs using the Bowtie alignment tool [23]. We identified 216 supercontigs that had ≥100 small RNAs, and which accounted for 52% of the genome size (10.8 Mb/20.8 Mb) and which contained 96% of the total small RNAs. Specifically, the small RNAs were highly enriched on 19 supercontigs that although only 0.4 Mb in total size contained ~50% of all sequenced small RNAs (Additional file 2: Table S1). As the structure of the genome is unknown at present, we do not know if the 19 supercontigs that were enriched in small RNAs belong to centromeric or telomeric regions. To analyze the overall small RNA density distribution on the genome, we scanned the genome using a 500 bp window, and counted numbers of small RNAs in each window. We defined a “hot spot” as containing ≥100 small RNAs per window; using these parameters, there were 784 “hot spot” windows out of 41,600 genomic windows. The graphic views of “hot spot” distribution further revealed that most small RNAs arose either from some large clusters (≥ 3 genes and up to 14 genes that have abundant small RNAs) (Additional file 1: Figure S4A) or from isolated peaks (Additional file 1: Figure S4B). There are regions of the genome that had only a few mapped small RNAs (Additional file 1: Figure S4C).

We analyzed the protein coding genes to which small RNAs mapped, and found that many genes had only a few small RNAs that mapped to them and hence are likely artifacts. Thus, we tested our dataset with different cutoffs for the number of small RNAs mapping to a gene; we used four measures (no cutoff, ≥10, ≥25 or ≥50 small RNAs mapping to a gene). For each cutoff, we identified the number of protein coding genes in each category (antisense only small RNAs; sense and antisense small RNAs; and sense only small RNAs) and plotted the microarray expression value for these genes (see later section on "A global assessment of genes potentially regulated by small RNAs in *E. histolytica*"). For the two least stringent criteria (no cutoff or ≥10 small RNAs mapping to a gene), we observed no significant difference in the microarray expression value for the three categories of protein coding genes (Additional file 1: Figure S5A and Additional file 1: Figure S5B). However, when we used either the ≥25 or ≥50 small RNA cutoff, we identified significantly lower expression among genes with antisense or sense/antisense small RNAs compared to genes with sense small RNAs (Additional file 1: Figure S5C and Additional file 1: Figure S5D). The total number of protein coding genes using either cutoff (≥25 and ≥50 small RNAs) was relatively similar (420 and 358 genes, respectively). To be as stringent as possible, we decided to use a cutoff of ≥50 small RNAs mapping to a gene for further analysis. Overall, 358 protein coding genes (~4% of the genome) had ≥50 small RNAs that mapped to them. These protein coding genes could be categorized into three groups: (I) 226 genes with only antisense small RNAs; (II) 45 genes with both antisense and sense small RNAs; and (III) 87 genes with only sense small RNAs. Most genes in group I and II are annotated as hypothetical proteins (206 out of 271). However, a few gene families were represented including AIG1 family proteins (28 genes), beta-amylase (8 genes), deoxyuridine 5^′^-triphosphate nucleotidohydrolase domain proteins (6 genes), DNA polymerase (5 genes), and C2 domain proteins (2 genes).

In order to determine whether protein coding genes with small RNAs are in proximity to each other, we characterized the patterns of genes to which small RNAs map. A cluster is defined as ≥3 contiguous genes (with ≥50 small RNAs mapping to each gene). A pair is defined as 2 contiguous genes (with ≥50 small RNAs mapping to each gene) that are ≤1000 bp apart. There are a total of 358 protein coding genes that have ≥50 small RNAs and of these the majority are in clusters (167 genes) or in pairs (46 genes). These clustered/paired genes were largely in group I and II categories. We next looked at transcript orientation in the paired genes. The 46 protein coding genes in pairs had 6 divergent pairs (defined as tail to tail), 7 convergent pairs (defined as head to head), and 10 tandem pairs (defined as two adjacent genes in the same orientation). A total of 145 protein coding genes are unpaired with 63 genes in group I, 8 genes in group II, and 74 genes in group III.

When we characterized clustered protein coding genes (in group of ≥3) with small RNAs, we identified 26 clusters, which ranged in size from a cluster of 3 genes to clusters as large as 14 genes (Additional file 2: Table S2). A substantial number (19 out of 26) of clusters are in previously identified regions of D1, D2 and D4 genome duplication, which are segmental duplications with each segment flanked on both ends by inverted repeats such as IR/EhERE1/EhLINEs [32]. Clusters that are not in regions of D1-D4 genome duplications are still flanked by repetitive elements at either one or both ends (Additional file 2: Table S2). Thus, clustered genes (that have large numbers of small RNAs) are more likely to be associated with repetitive elements.

In order to determine whether small RNAs are enriched on paired or clustered protein coding genes or in the intergenic DNA regions, we calculated the small RNA density on these paired/clustered genes as well as the intergenic regions between genes. This was calculated as small RNA/bp. We identified that the density of small RNAs mapping to intergenic regions was significantly lower compared to small RNA density mapping to paired/clustered genes (Additional file 1: Figure S6)(mean value 0.12 vs 0.54; p-value < 2.2e-16). This indicates that small RNA synthesis is most likely templated using a given gene rather than a long template covering several genes. For intergenic regions that had high small RNA density, we found these small RNAs are often in discrete sections or adjacent to predicted genes. Thus, we postulate that this may be due to small RNAs mapping to an unannotated gene or UTRs.

In summary, our analysis suggests that the small RNA targeted protein coding genes tend to be in pairs or clusters, and that clusters of genes with small RNAs are more often associated with repetitive elements. Small RNA density on paired/clustered genes versus intergenic regions implies that it is unlikely for either DNA or a long transcript covering several genes to be used as a template, but rather that transcript derived from each gene is the most likely template.

### Small RNA distribution patterns within protein coding genes

**Figure 2:**
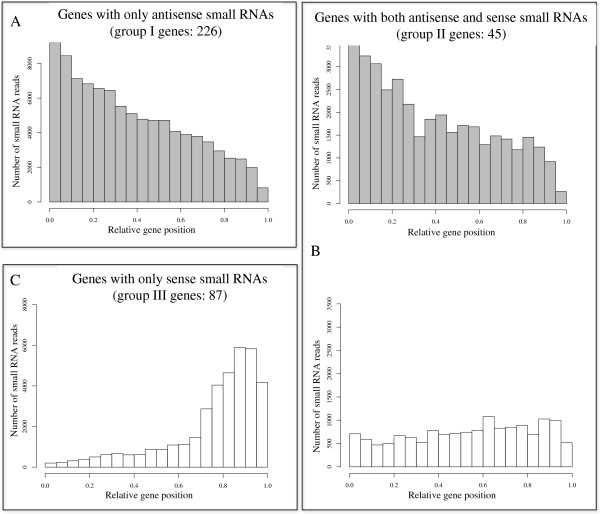
**Genome-wide analysis of small RNA distribution patterns to amebic protein coding genes.** Each small RNA read was assigned a position value based on the position of the starting nucleotide along the gene. The gene length for each protein coding gene was normalized to one. Total number of small RNA reads (y-axis) was plotted from 5^′^ to 3^′^ according to their relative position within all genes (x-axis). (**A**) Small RNA distribution on genes with only antisense small RNAs. (**B**) Small RNA distribution on genes with both antisense (gray) and sense small (white) RNAs. (**C**) Small RNA distribution on genes with only sense small RNAs. Grey bars: antisense small RNAs; White bars: sense small RNAs.

We have previously shown that small RNAs that map antisense to protein coding genes tend to be most abundant toward the 5^′^-end of genes [11]. However, that analysis was done using a very limited dataset of small RNAs generated from Sanger sequencing. Our new pyrophosphate sequencing dataset enabled us to examine this observation on a larger scale. Using the stringent criteria of ≥50 small RNAs mapping to a gene, a total of 226 protein coding genes were categorized as group I (genes with only antisense small RNAs). We plotted small RNA distribution along each gene (normalizing the gene length to one; with the position of each small RNA determined by its first nucleotide within the mapped protein coding gene) (Figure 2A). There was a clear trend showing that most antisense small RNAs mapped toward the 5^′^-termini of predicted genes. This trend holds true for most targeted protein coding genes (174/226 or 77%) and was not caused by a few genes with a high number of small RNAs at the 5^′^-end. For the 5^′^-polyP small RNAs in *Ascaris* and *C. elegans*, there is a clear difference of small RNA distribution on their corresponding mRNA targets. In *C. elegans* 22G-RNAs are mostly enriched at 3^′^-end of the mRNA [33], while *Ascaris* 22G-RNAs are distributed toward the 5^′^-end of mRNAs [30]. Our results indicate that the small RNA distribution pattern for the antisense small RNAs in *E. histolytica* is more similar to that in *Ascaris* as compared to *C. elegans*. The 5^′^-bias of antisense small RNAs could reflect the heavy recruitment of RdRP complexes to these regions, and the exact mechanism of RdRP in generating secondary antisense small RNAs is largely unknown at present.

**Figure 3:**
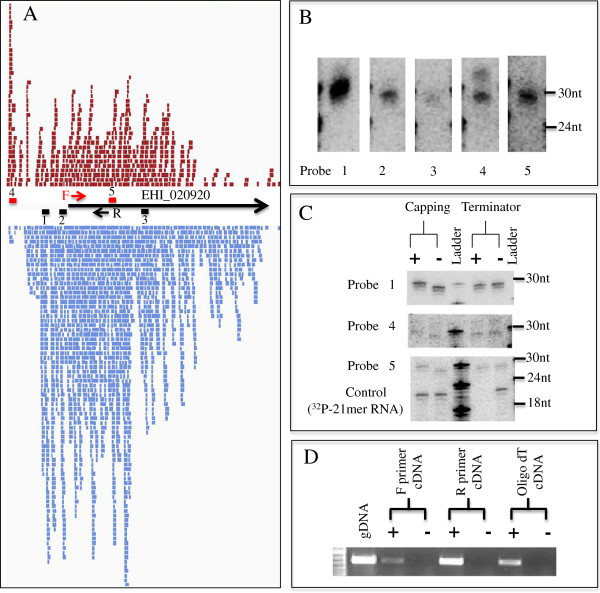
**Small RNA distribution and biochemical analysis of small RNAs for genes with both antisense and sense small RNAs. **(**A**) EHI_020920 (represented by arrow) is depicted with the small RNAs that map to it; each bar represents one unique small RNA (red: small RNAs map to upper strand, sense to EHI_020920; blue: small RNAs map to lower strand, antisense to EHI_020920). The positions of selected probes used for small RNA Northern blot analysis are represented by bars and numbers (black for detecting antisense small RNAs; red for detecting sense small RNAs). The positions of the F and R primers, used to generate cDNA for the strand-specific RT-PCR, are shown. (**B**) Northern blot analysis detected signal for antisense small RNAs (probes 1, 2, 3) and sense small RNAs (probes 4, 5). (**C**) Sense small RNAs have 5^′^-polyP termini. 10 μg small RNA enriched sample was treated with Terminator or Capping enzyme. Probes 1, 4, 5 were used for Northern blot analysis. A control oligonucleotide that is labeled with a 5^′^-monoP is degraded by Terminator and has no change in size with capping enzyme, as expected. (**D**) Strand specific RT-PCR demonstrates both sense and antisense transcripts for EHI_020920. cDNA was generated using F and R primer (to detect antisense and sense transcript, respectively) as well as oligo dT primer. RT-PCR reveals both antisense and sense transcripts with antisense transcript at lower abundance than sense transcript. Both RT (+) and control reactions lacking RT (−) are shown.

For the group II genes (45 genes with ≥50 antisense and ≥50 sense small RNAs), we similarly plotted the small RNA distribution along each gene. We noted that antisense small RNAs were distributed with a 5^′^-enrichment (42/45 genes or 93%), while the sense small RNA distribution pattern was more heterogeneous (5^′^-enriched in 16/45 genes; 3^′^-enriched in 20/45 genes; evenly distributed in 9/45 genes) (Figure 2B). Additionally, we noted that for group II genes, in most cases the number of antisense small RNAs was greater than the number of sense small RNAs for each gene locus (Figures 2B and 3A).

In contrast, when small RNA distribution was plotted for group III genes (87 genes with only sense small RNAs), it became apparent that sense small RNAs were enriched towards the 3^′^-end of each gene (81/87 or 93%) (Figure 2C). We noticed that genes with only sense small RNAs mainly code for four highly expressed gene families (i.e. 40S ribosomal protein S16 (4 genes); Gal/GalNAc lectin (6 genes); three protein kinase families (44 genes); and hypothetical proteins (4 genes)). Since our cloning method will capture all types of small RNAs (5^′^-cap, 5^′^-PPP, 5^′^-P, 5^′^-OH), degradation products with 5^′^-OH species would also be cloned; this may suggest that at least some of the sense small RNAs might be mRNA degradation products. However, we found no sense small RNAs to other highly expressed genes, indicating some specificity for sense small RNAs to these specific loci. Since the mapping of sense small RNAs to many genes often had abrupt boundaries within/near 3^′^ end of genes and sometimes extended into intergenic regions (Additional file 1: Figure S7), we felt that some mapping artifacts could be due to poor genome quality in these regions. As the *E. histolytica* genome is not complete at present, we cannot rule out whether or not these sense small RNAs are derived from some other loci.

Although sense small RNAs have been identified in almost all cloning libraries, these types of small RNAs have largely been ignored since it is hard to evaluate their identity as true endogenous small RNAs versus non-specific hydrolysis fragments. In both *C. elegans* and *Ascaris suum*, where 5^′^-polyP small RNAs were found, sense small RNAs were identified but were not characterized [30,33]. For the *E. histolytica* group III genes that have only sense small RNAs mapped to them we have not made any assumptions and have not further characterized the structure, derivation, or function of this category of sense small RNAs.

### Antisense and sense small RNAs (for group I and II genes) have 5^′^-polyphosphate termini

The secondary 5^′^-polyP small RNAs in nematodes (both *C. elegans* and *Ascaris suum*) are thought to be generated by an amplified gene silencing mechanism, likely through RdRP [30,33]. We have previously demonstrated that in *E. histolytica* both endogenous and exogenously triggered antisense small RNAs have 5^′^-polyP termini [11,12]. Since our analysis to date had focused on the antisense small RNAs, we wanted to determine whether the small RNAs that map sense to genes (in the group II category) also have 5^′^-polyP termini. For this purpose, we analyzed small RNAs that mapped sense to the EHI_020920 locus. This gene is highly enriched for both antisense and sense small RNAs with small RNA coverage extending into the apparent 5^′^-upstream region (Figure 3A). Northern blot analysis detected signal at 27nt with probes that detect antisense small RNAs (probes 1, 2 and 3) and with probes that detect sense small RNAs (probes 4 and 5) (Figure 3B). All the blots were performed using the same membrane, and therefore the relative band intensity reflects the abundance of small RNAs. We observed a correlation of 5'-enriched distribution of small RNAs with the intensity of probe 1 > probe 2 > probe 3. We then performed a Terminator exonuclease assay and a 5^′^-end Capping assay on the total RNA sample. The signal for sense small RNAs (probes 4 and 5) were resistant to Terminator treatment and shifted up after capping assay, indicating that these small RNAs that map sense to the EHI_020920 locus have 5^′^-polyP termini (Figure 3C). Probe 1, which would detect antisense small RNAs had the expected biochemical features consistent with 5^′^-polyP termini. The control (a “spiked” synthetic 21-mer RNA species, which was labeled to have a 5^′^-monophosphate structure) was, as expected, degraded by Terminator enzyme and unaffected by treatment with capping enzyme.

We further examined a second example: the locus (DS571502:1400–2800 bp) that contains both antisense and sense small RNAs to EHI_130480 and EHI_130490 as well as one potential unannotated gene (Additional file 1: Figure S8). Probes for detecting antisense (probe 7) and sense (probe 6) small RNAs were chosen for EHI_130480. Northern blot analysis showed signal for both probes at ~27nt; the sense small RNA was resistant to Terminator exonuclease and thus has 5^′^-polyphosphate termini. Thus, for the two loci tested (both of which had abundant antisense and sense small RNAs) we determined that the sense small RNAs also have 5^′^-polyP termini.

The biochemical features of sense small RNAs (in group II genes) having 5^′^-polyP termini was unexpected. Analysis for pairing between antisense and sense small RNAs showed no enriched pairs for these gene loci (data not shown). In a typical RNAi pathway, dsRNA is chopped into a siRNA duplex, where enriched pairing between antisense and sense small RNAs can be found [34,35]. The lack of pairing between antisense and sense small RNAs in group II genes and the 5^′^-polyP termini for both antisense and sense small RNAs indicates that these small RNAs could be individually processed from bidirectional transcripts at these loci. To determine if this may have occurred, we performed strand-specific RT-PCR for EHI_020920, EHI_130480 and EHI_130490. At all three loci, both sense and antisense transcripts can be detected, albeit with antisense transcripts at much lower level than the sense transcript (Figure 3D, Additional file 1: Figure S8C). Overall transcript levels as assayed by RT-PCR correlated with the abundance of antisense and sense small RNAs mapped to these loci. We further applied the strand-specific RT-PCR assay to two additional protein coding genes (EHI_192590 and EHI_148800), which had both antisense and sense small RNAs and identified transcripts in both directions for these loci (data not shown). Thus, we conclude that although bidirectional transcription has not been previously documented in *E. histolytica*, both antisense and sense small RNAs are likely templated from bidirectional transcripts and generated by an RdRP-dependent mechanism resulting in small RNAs with 5^′^-polyphosphate termini. Natural antisense transcripts have been shown to be a major source of siRNA generation in *Drosophila melanogaster*[36] and in plants [37]. In the parasite, *Giardia lamblia*, bidirectional transcription produces abundant sterile antisense transcripts [38]. Further studies to characterize the extent of natural antisense transcripts in *E. histolytica* and the mechanism for generation of 5^′^-polyP small RNAs are needed. Our demonstration that small RNAs to group II genes (genes with both antisense and sense small RNAs) have 5^′^-polyP termini and strand-specific RT-PCR detecting transcripts in both directions indicates that these sense small RNAs are likely generated from antisense transcript, and indicates that some portion of “sense” small RNAs in our dataset although called “sense”, are truly antisense to the transcript derived from the opposite strand of the examined gene.

### Small RNAs are derived from both unspliced and spliced transcripts

In order to identify whether small RNAs map to spliced or unspliced transcripts, we downloaded both genomic and mRNA sequences for all *E. histolytica* protein coding genes with at least one predicted intron. Small RNAs mapping to introns are defined as those that map to the genomic gene sequence but not the mRNA sequence. Small RNAs mapping to exon-exon junctions are those that map to mRNA sequence but not the genomic gene sequence. We found a total of 52 small RNA reads that spanned exon-exon junctions (51 mapped in the antisense orientation and 1 mapped in the sense orientation) and 1,187 small RNA reads that mapped to predicted introns (908 mapped in the antisense orientation and 279 mapped in the sense orientation) (Additional file 2: Table S3).

We further examined the protein coding genes with at least 50 small RNAs and which also have at least one intron (Additional file 3: Table S8). For this list, we first checked for potential “false introns” caused by genome sequence error, and excluded EHI_018150, EHI_180820 and EHI_137120 as they have many Ns in their intron sequences. We then checked the predicted intronic sequence in the remaining genes for an in-frame stop codon or frame disruption, as this strongly suggests that the intron is correctly predicted (Additional file 3: Table S8). Lastly, we checked for paralogs within these genes. With these criteria, we examined the three categories of genes to which small RNAs mapped to identify small RNAs that mapped to exons, introns and exon-exon junctions (Additional file 3: Table S8). For the group I genes, 18 unique genes had small RNAs that mapped to predicted introns and among them, 4 unique genes also had small RNAs that mapped to exon-exon junctions. For the group II genes, 4 unique genes had antisense small RNAs that mapped to introns and 3 of these also had sense small RNAs that mapped to introns. None of the genes in this category had small RNAs that mapped to exon-exon junctions. For the group III genes, 4 unique genes had small RNAs mapped to predicted introns, but none mapped to exon-exon junctions. Overall, we made a number of observations: (i) a greater number of small RNAs mapped to exons than to the introns for all three groups of genes; (ii) all but one intron-containing gene in groups I and II had small RNAs that mapped to introns; and (iii) only a limited number of genes (4 unique genes) had small RNAs that mapped to exon-exon junctions. These data indicate that both spliced and unspliced transcripts are capable of being used as templates to produce small RNAs in *E. histolytica.* As an example, the mapping of small RNAs to exons, introns and exon-exon junctions are shown for EHI_135940 and EHI_197360 genes (Additional file 1: Figure S9). Further calculations of the small RNA density revealed four-fold greater density of small RNAs in exons than in introns [EHI_135940, exon (0.77) vs. intron (0.19); EHI_197360, exon (0.39) vs. intron (0.10)] (Additional file 2: Table S4). The difference could suggest that spliced transcripts are preferred as templates to unspliced transcripts, or alternatively may simply be a reflection of the ratio of spliced and unspliced transcripts available in the cell.

In *C. elegans,* EGO-1, an RdRP, is critical for *C. elegans* germline development and is responsible for producing 5^′^-polyP antisense small RNAs from mRNA-derived loci. Small RNA sequencing has shown that small RNAs often span exon-exon junctions and rarely map to introns, indicating EGO-1 uses processed mRNA as a template [39]. RdRP could theoretically template on genomic DNA, nascent transcripts, or processed mRNAs. Small RNAs that map to exon-exon junctions provide evidence that a spliced mRNA template is used to generate these small RNAs, whereas small RNAs that map to introns indicate that non-spliced templates can also be used to generate small RNAs. Based on the observations that there are small RNA free genomic regions between genes with antisense small RNAs, and that many more small RNAs map to exons than introns, we conclude that the *E. histolytica* RNAi machinery prefers mature transcript as a template for generating small RNAs. However, the machinery in *E. histolytica* also seems capable of using unspliced transcripts as template, although at reduced levels. Whether this is indicative of the inherent preference of the *E. histolytica* machinery or due instead to the low abundance of unspliced mRNA is not clear at present.

### Small RNAs that map to tRNAs, rRNAs and retrotransposon elements

In order to identify small RNAs that map to the tRNAs, rRNAs and retrotransposon elements, we followed the outline in Additional file 1: Figure S1. *E. histolytica* has uniquely organized tRNA genes that are in multiple tandem-array units, likely arranged at subtelomeric regions and spaced by tandem repeats of AT-rich sequences [40]. The *E. histolytica* rRNA genes reside on an extrachromosomal circular plasmid and two rRNA transcription units are organized as inverted repeats [41]. We mapped the small RNA reads to the tRNA repeat units and the rRNA plasmid. We found that nearly all small RNA reads were in the sense orientation to the coded tRNAs and rRNAs. Additionally, we plotted the size distribution and nucleotide frequency for rRNA reads and noted that small RNAs in these categories did not peak at 27nt (Additional file 1: Figure S10), and did not have a 5^′^-G enrichment (Additional file 1: Figure S11) indicating that these small RNAs are most likely degradation products resulting from these highly expressed structural RNAs. However, we are aware of some recent reports showing that small RNAs could originate from tRNAs and snoRNAs [42,43]. Upon a closer examination of the tRNA reads, we noticed a slight peak at 27nt with some degree of 5^′^-G enrichment for this 27nt population only (Additional file 1: Figure S10 and Additional file 1: Figure S11). Whether or not this indicates that these are functional small RNAs in *Entamoeba* needs further study.

Transposons and repetitive DNA are abundant in *Entamoeba* and hundreds of copies of the long interspersed nuclear elements (LINEs) and short interspersed nuclear elements (SINEs) can be found in the *E. histolytica* genome [44,45]. Our small RNA dataset contains 5% of reads that mapped to LINE and SINE elements. Analysis of the lengths of these small RNAs showed two peaks (one at 27nt and the other at 17nt) (Additional file 1: Figure S10). When nucleotide composition is plotted, the 5^′^-G propensity is apparent for the 27nt peak, but not for the 17nt peak (Additional file 1: Figure S12). Thus, the 27nt small RNA population that maps to LINE/SINE elements had features similar to those that map to coding regions and are likely not artifacts. As an example, we mapped small RNAs to the EhRLE5 sequence, which has been categorized in the EhLINE1 family [46]. The small RNAs are scattered along the whole region on both strands and cover the whole EhRLE unit, with a slight increase in small RNAs near each end (Additional file 1: Figure S13). We got a positive signal from Northern blot analysis using several probes to retrotransposon elements although the size by Northern blot analysis was slightly higher (~32nt) than the cloned small RNA. This indicates that small RNAs could derive from these retrotransposon elements.

The mapping of small RNAs to D1-D4 repetitive segments showed a large number of small RNA reads on D1, D2 and D4 segments but not on D3 segments (Additional file 2: Table S2). Annotated protein coding genes in these duplication regions appear to be covered by large numbers of antisense small RNAs, forming a large cluster (Additional file 1: Figure S14 and Additional file 2: Table S2). Thus, although the overall numbers of small RNAs that associate with EhAGO2-2 and map to repeat or retrotransposon elements is low, they may play a functional role in controlling genome stability as has been shown in other systems [47,48]. Alternatively, small RNAs may have a role in controlling these retrotransposon elements but may do so by associating with the two other Argonaute proteins in *E. histolytica*.

### A global assessment of genes potentially regulated by small RNAs in *E. histolytica*

**Figure 4:**
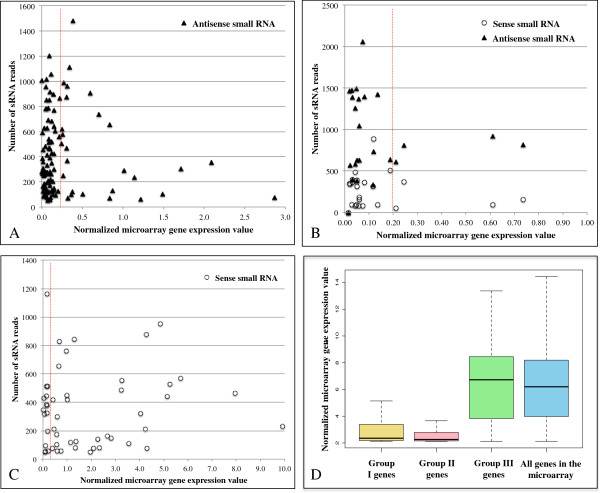
**Correlation between microarray gene expression values and the number of small RNAs targeting to a gene.** Genes are plotted using the number of mapped small RNAs (y-axis) and their normalized microarray expression levels (x-axis). The dashed line indicates the cutoff (0.2) for gene expression generally detectable by RT-PCR. (**A**) Plot for group I genes (each gene is represented by solid black triangle). (**B**) Plot for group II genes (each gene is represented by solid black triangle for antisense small RNA and open circle for sense small RNA). (**C**) Plot for group III genes (each gene is represented by an open circle for sense small RNA). (**D**) Box-and-whisker expression plots for genes in groups I, II, III as well as all genes represented on the microarray. The top and bottom ends of each box represent the 75th and 25th percentile, respectively; the middle line represents the median value.

We have previously shown an inverse correlation between gene expression and antisense small RNA abundance raising the intriguing possibility that antisense small RNAs may mediate target gene silencing in *E. histolytica*[11]. However, those data were on a very limited scale due to the very limited set of sequenced small RNAs. The pyrosequencing dataset allowed us to assess the potential genome-wide affects of these small RNAs by comparing small RNA abundance with microarray expression data. We used previously published microarray data from *E. histolytica* HM-1:IMSS trophozoites (the same strain from which the small RNA library was generated) [49]. The analysis was conducted for the three groups of protein coding genes with distinct small RNA mapping patterns. For group I genes (≥50 small RNAs that mapped antisense to the gene), there were 226 protein coding genes that met the criteria; of these, 116 genes are represented on the microarray. We plotted the number of mapped small RNA reads for each gene as a function of normalized microarray expression data and identified that most genes (90) in this category are not expressed (Figure 4A and 4D). For group II genes (≥50 small RNAs in both the antisense and sense orientation) there are 45 genes that met our criteria and 26 are represented on the microarray and most of these genes (22) are also not expressed (Figure 4B and 4D). In order to determine whether these 112 genes (that have antisense or sense/antisense small RNAs and are not expressed in *E. histolytica* trophozoites under standard conditions) are expressed under other conditions, we compared the expression profiles of these genes across all other conditions tested (various *E. histolytica* strains, stress conditions, culture conditions, stage conversion) [49-53]. Of these 112 genes, 30 were expressed in a strain-specific manner, 35 were regulated under various culture conditions, and 55 were regulated during stage conversion (some genes changed in multiple array conditions) (Additional file 2: Table S5). Overall, a total of 270 protein coding genes are not expressed in *E. histolytica* HM-1:IMSS trophozoites under standard *in vitro* growth conditions [49]. Based on our sequencing data we estimated that small RNAs target about 41% of these protein coding genes (112 out of 270 genes). Of these 112 genes, a large proportion (95 genes) change in expression profile under one or more conditions examined indicating that they are not permanently silenced and may be regulated by small RNAs.

For the genes in group III (≥50 mapped sense small RNAs), 87 genes met our criteria with 64 represented on the microarray. Plotting the number of mapped reads for each gene as a function of its normalized microarray gene expression value showed no direct link between numbers of sense small RNAs to a gene and the expression level of that gene (Figures 4C and 4D). We reasoned that for genes with high expression value, the sense small RNAs may represent degradation products. However, for genes with very low mRNA expression (15 of the 87 on the microarray), we have no good explanation on how these sense small RNAs were generated. Whether this represents an artifact of errors in genome assembly or some other factor is not clear at present. Interestingly, none of these genes are regulated under other conditions tested (data not shown).

In summary, the strongest correlation between gene expression and small RNA abundance was for genes that had either abundant antisense small RNAs or abundant sense and antisense small RNAs. Although simply correlative at present, the inverse correlation between antisense small RNA abundance and gene expression suggests that antisense small RNAs mediate target gene silencing in *E. histolytica* and are potentially involved in gene regulation under various conditions.

### Small RNA sequencing from the nonvirulent *E. histolytica* Rahman strain

It has been observed that pathogenicity varies greatly among different *E. histolytica* strains. *E. histolytica* HM-1:IMSS is a virulent strain, while *E. histolytica* Rahman is a nonvirulent strain [54]. We have previously demonstrated that gene expression profiles are substantially different between these two strains and have used the distinct strain-specific expression profiles to identify virulence genes [55]. Given the data above, and to explore whether small RNAs play a role in strain-specific and/or virulence gene regulation, we constructed a small RNA library from trophozoites of the *E. histolytica* Rahman strain.

We visualized the small RNA populations in *E. histolytica* Rahman by separating total RNA on a 12% denaturing polyacrylamide gel followed by Sybr gold staining and visualized an abundant 27nt small RNA population (data not shown). A small RNA library was constructed from size-fractionated RNA (15-30nt) using a 5^′^-P independent cloning approach and limited pyrophosphate sequencing was performed generating 151,656 reads. For the purpose of mapping, we used *E. histolytica* HM-1:IMSS genome as a reference genome rather than the current *E. histolytica* Rahman assembly, based on the following facts: (a) the current *E. histolytica* Rahman genome assembly (Amoeba DB, http://amoebadb.org) is in a preliminary stage containing 17,378 small contigs and is unannotated, (b) there is a high similarity between these two strains and one previous study has estimated that only 5 out of a sample of 1,817 (0.3%) genes were identified as highly or significantly divergent [56], and (c) Affymetrix platform microarrays found no difference in overall hybridization efficiency levels compared to HM-1:IMSS (i.e. the signal scale factor was similar between two arrays and thus both RNA samples hybridized efficiently to the array chip), indicating a high level of sequence identity for the protein coding genes [49]. We realize that sequence differences between the *E. histolytica* HM-1:IMSS and Rahman strains may cause us to lose some data. However, the advantages of being able to map to an annotated genome and thus determine how many small RNAs map to protein coding genes (and whether they map sense or antisense) and to intergenic regions were significant enough that we proceeded with the data generated by aligning the *E. histolytica* Rahman small RNA library to the *E. histolytica* HM-1:IMSS genome sequence.

**Figure 5:**
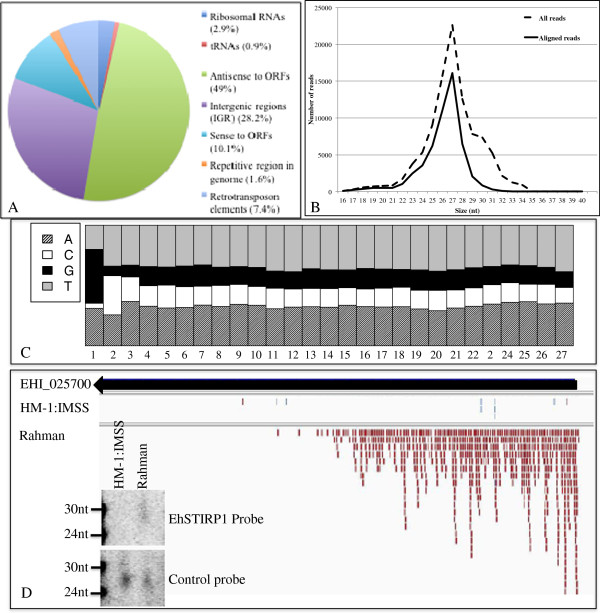
**Small RNA populations in the non-virulent *E. histolytica *Rahman strain. **(**A**) Distribution of small RNA reads cloned from a size-fractionated small RNA library from the non-virulent *E. histolytica* Rahman strain. (**B**) Small RNA length distribution for reads in the Rahman library (dashed black line: all reads after filtering out those that originate from structural RNAs and repetitive elements reads; solid black line: reads that align to the reference genome). (**C**) Nucleotide frequency at each position for the aligned 27nt reads in the Rahman library reveals a 5^′^-G predominance. (**D**) Antisense small RNAs from the *E. histolytica* Rahman strain map to EhSTIRP1 (EHI_025700). Small RNA reads from HM-1:IMSS EhAGO2-2 IP library and Rahman size-fractionated small RNA library are shown in red bars (small RNAs in antisense direction) and blue bars (small RNAs in sense direction); black arrow represents EHI_025700 and its direction. Insert figures are Northern blot analysis using total RNA samples from both strains to show the presence of antisense small RNAs in the *E. histolytica* Rahman strain but not in HM-1:IMSS strain for selected EhSTIRP1 probe. Control probe (EHS-D-A16-1) detected small RNA signal in both samples.

Following the same small RNA sequence analysis flow-chart as applied to the *E. histolytica* HM-1:IMSS library, the *E. histolytica* Rahman dataset was analyzed (Additional file 1: Figure S1). Overall, there were 98,414 unique sequence reads, with 84.1% (82,780) of the sequences found to have been sequenced only once (Table 2 and Figure 5A). Small RNAs that mapped to tRNAs (517 reads), rRNAs (1,753 reads) and repetitive elements (EhSINEs/EhLINEs and EhERE elements) (4,439 reads) were subtracted from the dataset. The remaining reads were aligned to the *E. histolytica* HM-1:IMSS genome and to the predicted protein coding genes (52,028 reads mapped to the genome and 35,157 reads mapped to the protein coding genes) (Table 2). The mapping of *E. histolytica* Rahman small RNA dataset showed a similar overall distribution pattern as that of the small RNA EhAGO2-2 IP library: many small RNA reads from the Rahman library mapped antisense to genes (49%); the two other main categories of small RNAs were those that mapped sense to genes (10.1%) and to intergenic regions (28.2%) (Figure 5A). In addition, the size distribution of the aligned reads in Rahman showed a peak at 27nt (Figure 5B) with a 5^′^-G bias (Figure 5C). Furthermore, there was comparable sequence depth for the mapped 27nt reads between the HM-1:IMSS and Rahman libraries (Figure 1B and Figure 5B). Thus, given that many features are conserved between two libraries, we concluded that most reads from the Rahman size-selected library may be from AGO2-2 bound species.

**Table 2:** **The algorithm for small RNA processing for the *E. histolytica *Rahman size-fractionated small RNA library**

**Procedures**	**Output**
5^′^-P independent cloning (5^′^CIP+PNK method)	Rahman total RNA, size fractionation (15-30nt) library
Total reads by Pyrosequencing	151,656 reads
Primer trimming, size-limiting (15–40nt)	140,571 reads
Unique sequences (percentage)	98,414 unique sequences (82,780 cloned only once; 84.1%) (15,634 cloned more than once; 15.9%)
Scan for tRNA, rRNA	tRNA: 517 sequences rRNA:1,753 sequences
Scan for SINE/LINE, EhERE elements	4,439 sequences
Map to *E. histolytica* genome	**52,028 sequences**
Map to *E. histolytica* predicted ORFs	35,157 sequences

Half of a pyrophosphate sequencing run was performed. Unix tools were used to remove adaptor and linker sequences. The Bowtie alignment tool was used to scan for structural RNAs, repetitive elements and for the final genome mapping. We used *E. histolytica* HM-1:IMSS genome as a reference genome due to the *E. histolytica* Rahman sequence not being assembled. The numbers of reads that map to the genome sequence at each step are listed.

To be confident that our mapping using the HM-1:IMSS genome represented the overall picture for the Rahman strain, we also aligned these small RNA reads to the current Rahman assembly and compared the number of mapped reads. Aligning the Rahman small RNA dataset to the *E. histolytica* Rahman assembly rather than the HM-1:IMSS genome only increased the number of mapped reads by 7% (7,232 reads). Overall, this indicates that mapping of small RNAs from Rahman to the HM-1:IMSS genome is representative of the overall picture and the greater information gained by mapping the small RNAs to an annotated assembly outweighed the negative effects of using a genome sequence from a different strain.

### Genes with strain-specific expression patterns and roles in virulence have small RNAs that map to them

We cannot directly compare the HM-1:IMSS and Rahman libraries for read frequencies and patterns of small RNA mapping, as they were generated differently and were sequenced to different depths. However, common features in both libraries (peak at 27nt, 5^′^-G bias, and mapping antisense to genes) suggested that EhAGO2-2 bound species are represented in the Rahman library. Thus, this overlap of small RNA coverage between the two libraries allowed us to look for genes, which might be regulated strain specifically on the basis of small RNA abundance. An important caveat to the analysis is that while the presence of small RNAs is meaningful in either strain, the absence of small RNAs in the Rahman library is less meaningful and could be due to the limited sequencing depth or use of a size-selected small RNA library.

Using the same criteria as used for HM-1:IMSS (genes with ≥50 small RNAs that map to them) the small RNA library generated from *E. histolytica* Rahman identified 223 genes with small RNAs (175 genes with antisense small RNAs; 29 genes with both antisense and sense small RNAs; and 19 genes with only sense small RNAs). When the genes from these three categories were compared between the Rahman and HM-1:IMSS strains, we found significant overlap for genes with antisense and sense/antisense small RNAs. A total of 90 genes with only antisense small RNAs were common between the two strains (out of a total of 226 genes for HM-1:IMSS and 175 genes for Rahman); 16 genes with both antisense and sense small RNAs were common between the two strains (out of a total of 45 genes for HM-1:IMSS and 29 genes for Rahman). However, for the genes with sense only small RNAs, no overlap was identified (out of a total of 87 genes for HM-1:IMSS and 19 genes for Rahman). These data further support the idea that antisense small RNAs are likely playing roles in conferring strain-specific gene expression profiles, whereas the small RNAs that map sense only to genes are likely to be random degradation products.

In order to compare gene expression patterns for genes with strain-specific small RNAs, we used previously published microarray data for the two *E. histolytica* strains and a two-fold difference in normalized expression and a p-value <0.05 as cutoff [49]. Of the 160 genes that have lower expression in HM-1:IMSS than in the Rahman strain, 11 genes could be potentially regulated by antisense or antisense/sense small RNAs in HM-1:IMSS (these genes had small RNAs in the HM-1:IMSS strain but none in the Rahman strain). Of the 130 genes that have higher expression in the HM-1:IMSS strain compared to the Rahman strain, 19 genes could be potentially regulated by antisense or antisense/sense small RNAs in Rahman (these genes had small RNAs in the Rahman strain, but none in the HM-1:IMSS strain).

The *E. histolytica* serine, threonine, isoleucine, rich protein (EhSTIRP) was previously identified as a virulence determinant as it has high expression in *E. histolytica* virulent strains and no expression in nonvirulent strains and genetically proven to have a role in virulence [57]. There were 519 small RNAs that mapped antisense to this gene from our Rahman small RNA dataset compared to virtually no antisense small RNAs from the HM-1:IMSS small RNA dataset (Figure 5D). The distribution of the antisense small RNAs to EhSTIRP gene showed a clear enrichment at the 5^′^-end. We further confirmed the presence of EhSTIRP antisense small RNAs in the Rahman strain (and absence in the HM-1:IMSS strain) by Northern blot analysis (Figure 5D) using total RNA samples from two strains. Lastly, we attempted to overexpress a Myc-tagged EhSTIRP1 (EHI_025700), which resulted in successful EhSTIRP overexpression in the HM-1:IMSS strain but not in the Rahman strain even at very high levels of drug selection (data not shown). The lack of ability to overexpress the EhSTIRP gene in the *E. histolytica* Rahman strain that has abundant antisense small RNAs that map to it raises the intriguing possibility that the antisense small RNAs control the silencing of this virulence gene in the *E. histolytica* Rahman strain. Future studies will be needed to address this question.

## Discussion

In this study, we performed pyrosequencing of *E. histolytica* endogenous small RNAs that associate with the EhAGO2-2 protein in the virulent *E. histolytica* HM-1:IMSS strain and size selected small RNAs in the non-virulent *E. histolytica* Rahman strain. Characterization of these small RNAs showed that they are distinct in size (27nt), have preference for 5^′^-G, and have 5^′^-polyphosphate termini. Genome mapping revealed that these 27G-RNAs are mainly derived from coding genes with a much smaller population coming from retrotransposons. Comparison of the 27nt small RNA population from *E. histolytica* strains with varying virulence and expression profiles demonstrated an inverse correlation between antisense small RNA and gene expression levels, hinting that antisense 27G-small RNAs may be involved in the regulation of strain-specific genes, including known virulence determinants, such as EhSTIRP.

We have previously demonstrated that 27nt antisense small RNA in *E. histolytica* have 5^′^-polyP termini [11]. In this study, we further characterized small RNAs that associate with EhAGO2-2, including those small RNAs that map both antisense and sense to genes. For all the small RNAs that were detectable by Northern blot analysis, we were able to show that they have 5^′^-polyP termini, indicating these abundant small RNA species in *E. histolytica* are reminiscent of secondary small RNA from *C. elegans* and *Ascaris*[29,30]. In worms, the biogenesis and stability of 5^′^-polyphosphate small RNAs are dependent on RdRPs and WAGOs respectively, with these small RNAs being a component of the siRNA amplification pathway [33]. Signal amplification is controlled by using primary trigger siRNAs to instigate secondary siRNAs through RdRP for the enforced silencing, but limiting secondary siRNAs from doing further signal amplification [58]. Although small RNAs that mapped sense to coding regions were found in *C. elegans* and *Ascaris suum* 5'-monoP independent libraries, their existence and functionality were not confirmed; instead they were generally treated as non-specific degradation products [30,33]. We have confirmed that in *E. histolytica* small RNAs that map sense to genes (which also have antisense small RNAs) are detected in Northern blot analyses as discrete band and bear the same 5^′^-polyP termini. Strand-specific RT-PCRs detected transcripts in both directions for these loci (albeit at a lower level for the antisense transcript), implying that RdRP-based small RNA generation could occur for both sense and antisense transcripts in this parasite. The *E. histolytica* genome encodes one full RdRP gene (EHI_139420) and two genes with partial RdRP domains (EHI_179800 and EHI_086260). The functions of the RdRP genes in *E. histolytica* RNAi pathway are still elusive and need further investigation.

Due to the fact that gene knockout is not feasible in *E. histolytica* we have been unable to dissect how the parasite RNAi components affect the levels of these small RNAs. The comparison of 5^′^-polyP small RNAs among the three organisms in which they have been described (*C. elegans*, *Ascaris* and *E. histolytica*) show several differences: (a) 5^′^-polyP small RNA size in *E. histolytica* is 27nt, whereas in *C. elegans* and *Ascaris* these RNAs are 22nt; (b) the distribution pattern of antisense small RNAs to the targeted gene loci is enriched at the 5^′^-end for *E. histolytica* and *Ascaris* whereas in *C. elegans* there is enrichment at the 3^′^-end of transcripts; (c) localization of EhAGO2-2 and bound 27nt small RNAs are mostly localized to the parasite nucleus [12], whereas *C. elegans* 22G-RNAs can associate with several different WAGOs and have both perinuclear [33] and nuclear localization [59-61]; and (d) *C. elegans* strongly prefers spliced transcripts as RdRP template for generating 5^′^-polyP small RNAs whereas both mature and nascent transcripts appear to function as templates in *E. histolytica*. Future studies aimed at elucidating these different mechanisms are needed.

Our previous limited Sanger sequencing has shown that small RNAs in *E. histolytica* largely mapped to the coding genes [11]. Our pyrosequencing data further confirmed this mapping, which is in contrast to other parasitic systems *T. gondii, G. intestinalis* and *T. brucei* where the small RNAs are 5^′^ single phosphate and mostly derived from repetitive elements, retrotransposons [10,15,16]. The genome of *E. histolytica* contains hundreds of copies of LINE and SINE elements, with SINE elements actively transcribed and LINE1 transcript detected by Northern blot analysis [55,62]. How retrotransposons are controlled in ameba is not known. As noted in our small RNA dataset analysis, one way that the RNAi pathway could function in this parasite is to target the unwanted transposon insertion events where segments are flanked on both ends by inverted repeats (IR/EhERE1/EhLINEs) as many small RNAs map to these regions. Whether RNAi directly targets retrotransposons in ameba is still an open question. Further characterization of the other two Argonaute proteins (EhAGO2-1 and EhAGO2-3) will provide a complete picture of small RNA populations in ameba and their functions in retrotransposon silencing.

The small RNA sequencing from different strains (HM-1:IMSS and Rahman) clearly indicated that expression of a subset of genes, including the virulence factor EhSTIRP, appears to be controlled by small RNAs in a strain-specific manner. In order to determine direct effects of the small RNA repertoire on parasite pathogenesis, the next step will be to perform functional studies to demonstrate direct roles for these small RNAs in regulating strain-specific virulence gene expression.

## Conclusion

In summary, we present two pyrosequencing small RNA datasets from the parasite *E. histolytica*: one an EhAGO2-2 IP library from the virulent *E. histolytica* HM-1:IMSS strain and the other a size selected small RNA library from the non-virulent *E. histolytica* Rahman strain. Our analysis identified a number of new findings: (1) amebic 27nt small RNAs have 5^′^ G preference; (2) antisense small RNA targeted genes are in pairs or clustered and notably most of clusters are from segmental duplications D1, D2 and D4; (3) characterization of group II gene loci shows that both sense and antisense small RNAs have 5^′^-polyphosphate termini; (4) small RNAs mapping to introns and exon-exon junctions were found indicating that both spliced and unspliced mRNA can serve as the templates for small RNA production; (5) few small RNAs are found in intergenic regions between paired/clustered genes indicating that RNA transcript from each gene was used as template; (6) small RNAs targeting retrotransponsons have similar features to the small RNA targeting the mRNAs, but are not highly abundant; and (7) antisense small RNAs may contribute to differential gene expression between virulent and nonvirulent amebic strains including the known virulence gene EhSTIRP. Thus, the two small RNA datasets in this study will provide important data for the community to study small RNA-mediated gene regulation in this important human pathogen.

## Methods

### Parasite culture and RNA preparation

*Entamoeba histolytica* trophozoites (HM-1:IMSS and Rahman) were grown under standard conditions as previously published [11]. A transfectant cell line expressing N-terminal Myc tagged EhAGO2-2 was previously described [11] and was maintained at 24μg/ml G418. For isolation of RNA that immunoprecipitated (IP) with EhAGO2-2, anti-Myc antibody (Pierce) was incubated with parasite lysate (2 hours, 4ºC), washed twice with 1x IP solution and pelleted. RNA was then isolated using mirVANA kit (Ambion). Small RNA enriched material from *E. histolytica* Rahman strain was prepared according to the mirVANA kit protocol.

### Library construction and sequencing

Small RNA cloning was based on the previously published protocol of the 5^′^-phosphate independent cloning method (CIP + PNK method) [11]. For the EhAGO2-2 IP RNA library, RNA was extracted from the IP material, directly ligated to the 3^′^-adapter oligonucleotide, size fractionated and treated with CIP and PNK, and then ligated to the 5´-adapter oligonucleotide. For the Rahman small RNA library, 100 μg of small RNA enriched RNA was size-fractionated on a 12% TBE urea polyacrylamide gel, the 15-30nt fraction was excised, followed by 3^′^-adapter ligation, CIP + PNK treatment, and 5^′^-adapter ligation as above.

For both libraries, the final ligated RNA with both 5^′^- and 3´-adapters was converted to single-stranded cDNA using Superscript II reverse transcriptase (Invitrogen, CA, USA). The cDNA was PCR-amplified using 454 Primers (A and B) for 20 cycles, and resolved on a 4% low melting point agarose gel. The band at 100 bp was excised and purified, and then further heat-denatured and purified from a 6% PAGE-urea gel. After a second round of purification, the recovered DNA was resuspended in Qiagen elution buffer, and pipelined into the 454 sequencing procedure.

### Small RNA read processing and mapping

All sequencing reads were processed by first removing the linker from both ends; the resulting sequences were analyzed with Unix tools and unique sequences selected. The unique reads were mapped against the *E. histolytica* HM-1:IMSS genome, release 1.3, (http://amoebadb.org/amoeba/) using the program Bowtie (http://bowtie-bio.sourceforge.net/index.shtml) with parameters set as -v 1, -k 5. Mapped reads were visualized with the genome browser IGV (http://www.broadinstitute.org/igv/). To identify small RNAs that map to exon-exon junctions, TopHat (2.0.0 release 4/09/2012, http://tophat.cbcb.umd.edu) was used with the following parameters: -report-secondary-alignments -G Ehistolytica_AmoebaDB-1.3.gtf -i 20. For the scaffold view of mapped small RNAs, histograms were generated in R (http://cran.r-project.org/), using a window size of 500 bp to divide the scaffolds. For tRNA and rRNA analysis, we downloaded all tRNA array sequences from NCBI based on previously published analysis [40]. For repetitive element analysis, we made a custom dataset using all SINEs/LINEs/EhEREs coordinates recently deposited to AmoebaDB (personal communication, Omar Harb), then aligned all sequences with Bowtie using the parameters -v 2, -k 5. All sequences from both small RNA libraries have been deposited at AmoebaDB (http://amoebadb.org/amoeba/). The small RNA sequence data from this study have been submitted to the NCBI Gene Expression Omnibus (GEO) under accession number GSE43668.

### Nucleotide composition of small RNAs and distribution of small RNAs on gene loci

For each subset of small RNA populations the small RNA read sequences were extracted. The nucleotide composition at each position was counted using the R package ShortRead, (http://bioconductor.org/ packages/release/bioc/html/ShortRead.html) and frequency bar plots generated from these data. For small RNA distribution on gene loci, only genes with ≥ 50 small RNAs mapping were considered. Each small RNA sequence was assigned a position value based on the position of the starting nucleotide along the gene (normalized by gene length; each gene has a total length of 1). Histograms for these values were plotted in R.

### Small RNA Northern blot analysis

Small RNA Northern blot analysis was done as previously published [11]. Briefly, 20 μg - 50 μg of small RNA enriched samples were separated on a denaturing 12% polyacrylamide gel and transferred to a membrane. This was probed with end-labeled ^32^P-labeled oligonucleotides in perfectHyb buffer (Sigma) at 37°C and washed using low (2X SSC, 0.1% SDS at 37°C for 15 min) and medium (1X SSC, 0.1% SDS at 37°C for 15 min) stringency conditions. All probes used are listed in Additional file 2: Table S6.

### Small RNA molecular analyses

For enzymatic analyses of RNA material, assays were done as previously published [11]. Briefly, either 10 μg or 50 μg small RNA enriched RNA sample was spiked with a control sample (a synthetic 21-mer RNA with the 5^′^-end labeled with ^32^P). For the Terminator assay, the sample mixture was treated with Terminator enzyme (Epicentre), following the provided protocol from the manufacturer. For the capping assay, the ScriptCap m^7^G capping system (Epicentre) was used with the alternate cap zero capping protocol. After enzymatic treatment, samples were phenol:chloroform extracted and resolved on a 12% polyacrylamide gel. Northern blot analysis was performed using a radiolabeled probe to detect the small RNA of interest.

### Strand specific RT-PCR analyses

We used SuperScript III first-strand synthesis kit (Invitrogen) for strand-specific cDNA synthesis and PCR analysis. *E. histolytica* HM-1:IMSS total RNA was treated with DNase I, and purified strand-specific primer for each gene (or oligo dT primer) was added to 0.5 μg total RNA reaction and heated to 65°C for 5 min. The temperature was lowered to 55°C, and prewarmed cDNA synthesis mix was added to the reaction and incubated at 55°C for 50 min. The reaction was terminated at 85°C for 5 min, chilled on ice, and 1 μl of RNase H was added to each tube and incubated for 20 min at 37°C before proceeding to PCR. In each primer reaction, both + RT and –RT reactions were performed, and the final cDNA volume was 20 μl. PCR was performed using 1 μl cDNA for a 30 μl PCR reaction for 33 cycles (94°C 15''; 55°C 30''; 72°C 1 min). Half of the volume of PCR reaction was loaded on a gel for visualization. Primers used are listed in Additional file 2: Table S7.

## Competing interests

No competing interests are declared by the authors.

## Authors’ contributions

HZ participated in the design of the study, performed experiments, analyzed the data and drafted the manuscript. GE performed the bioinformatics analysis and gave input on the manuscript. NH performed the pyrosequencing run and gave input on the manuscript. US conceived the study, participated in its design and coordination and edited the manuscript. All authors have read and approved the final manuscript.

## Supplementary Material

Additional file 1: Figure S1 Flow-chart for small RNA sequence analysis. The pipeline for processing of the small RNA sequences is listed. **Figure S2.** The number of loci to which each small RNA maps. The genome mapping file for the *E. histolytica* HM-1:IMSS small RNA dataset was used to generate the mapping counts for each small RNA in R using base functions. The number of small RNA reads (y-axis) is plotted against counts of their mapped loci (x-axis). **Figure S3.** Nucleotide frequency at each position for the 17nt, 26nt and 28nt small RNA sequences. A 5^′^-G sequence predominance is evident for the aligned 26nt and 28nt reads but not for 17nt reads when the nucleotide frequency at each position is plotted. **Figure S4.** Representative supercontig view of the mapped small RNAs. Small RNAs were binned into windows of 500 bp along the supercontig. The counts of small RNA reads (y-axis) were plotted against a normalized supercontig length of one (x-axis). Three major patterns were seen for the graphs of the binned distributions. **(A)** Abundant small RNAs from clusters with several hot areas; these are mostly for the 19 supercontigs with ≥5000 small RNAs. **(B)** Small RNAs largely confined to isolated peaks in supercontigs. **(C)** Very low numbers of small RNAs in a given supercontig. **Figure S5.** Expression of protein coding genes with mapped small RNAs, using different cutoffs (no cutoff, ≥10, ≥25 and ≥50 small RNAs mapping to the gene). We plotted the microarray expression value for three sets of protein coding genes: those with only antisense small RNAs (AS only); those with both antisense and sense small RNAs (AS + S); those with only sense small RNAs (S only). Using both the ≥25 and ≥50 small RNA cutoffs, we observed significantly lower expression values among genes with AS or AS + S small RNAs. The number of genes for each category are listed. **Figure S6.** The density of small RNAs on paired or clustered genes and associated intergenic regions. Box-and-whisker plots showing small RNA density (small RNA/bp) on paired or clustered genes vs. intergenic regions between genes. The top and bottom ends of each box represent the 75th and 25th percentile, respectively; the middle line represents the median value 0.54 (paired genes) vs. 0.12 (intergenic regions), p-value < 2.2e-16. **Figure S7.** Unusual mapping patterns for protein coding genes with only sense small RNAs. Genome browser view for EHI_189510 and EHI_070670, showing sense small RNAs as either having an abrupt boundary (EHI_189510) or crossing into the adjacent intergenic region (EHI_070670). Black arrow represents the predicted gene, red and blue bars represent mapped small RNAs; both are sense to genes. **Figure S8.** Biochemical analysis of small RNAs for genes with both antisense and sense small RNAs. (A) Antisense and sense small RNAs mapped to a region containing two annotated genes (EHI_130480 and EHI_130490, arrows) and one potential unannotated gene (red: small RNA mapped to upper strand, blue: small RNA mapped to lower strand). Probes for Northern blot analysis are represented by bars and numbers (black for detecting sense and red for detecting antisense to EHI_130480). **(B)** Northern blot analysis for small RNAs. Northern blot analysis detects antisense (probe 7) and sense (probe 6) small RNAs. The sense small RNA is resistant to Terminator cleavage assay, indicating that it does not have a 5^′^-monoP structure. **(C)** Strand-specific RT-PCR detects both sense and antisense transcript for both EHI_130480 and EHI_130490. cDNA was generated using F and R primer (to detect antisense and sense transcript, respectively) as well as oligo dT primer. RT-PCR reveals both antisense and sense transcripts with antisense transcript at lower abundance than sense transcript. **Figure S9.** Examples of antisense small RNAs found at both exon-exon junctions and introns to the same gene. Genome browser view for EHI_197360 and EHI_135940, showing antisense small RNAs can map both to introns and exon-exon junctions of the same gene. Black arrow represents the predicted gene, with their exons represented by blue bars. Red bars represent mapped small RNAs with direction from left to right (antisense to both genes). Green arrows point to introns, and exon-exon junction small RNA reads are broken red bars connected with lines. **Figure S10.** Small RNA size distribution for small RNAs mapping to structural RNAs and repetitive elements. The small RNA length distributions for small RNAs that map to tRNAs (grey), rRNAs (red) and repetitive elements (blue) are shown. A 27nt peak is evident for repetitive element reads but not for the structural RNAs. The “tailed” 17nt peaks seen for all three plots are most likely non-specific degradation from highly expressed transcripts. **Figure S11.** Nucleotide frequency at each position for the 17nt tRNAs and rRNAs, and the 27nt tRNAs. Nucleotide frequency at each position was plotted: no 5^′^-G sequence predominance was observed for 17nt rRNAs and 17nt tRNAs; a slight 5^′^-G enrichment was observed for 27nt tRNAs. **Figure S12.** Nucleotide frequency at each position of LINEs/SINEs mapped 17nt and 27nt sequences. Nucleotide frequency at each position was plotted for the 17nt and 27nt LINE/SINE sequences. There is a clear 5^′^-G sequence predominance observed for the 27nt sequences, but not for the 17nt sequences. **Figure S13.** Small RNAs mapped to EhLINE1 and Northern blot analysis. The EhRLE5 sequence, which belongs to the EhLINE1 family is used as an example to show small RNAs that map to repetitive elements. Upper panel: red, small RNA mapped to upper strand; blue, small RNA mapped to lower strand; long arrow, the complete EHRLE unit with arrow showing the RT transcription direction. The black bar is the position of selected EhRLE5 probe. Lower panel: Northern blot analysis revealed distinct bands at ~30nt size using probes selected for EhRLE5 and one locus of EHLINE1 (DS571716:427–2922). **Figure S14.** Small RNA mapping to genome duplication segment D1. Genome browser view for genome duplication segment D1 (red, small RNAs mapped to upper strand; blue, small RNAs mapped to lower strand). Annotated genes are shown below as dark blue blocks. All annotated genes are mapped with dense small RNAs in this scaffold indicating that the whole segment D1 might be a target of the RNAi pathway. Click here for file

Additional file 2: Table S1 List of top 19 supercontigs that are highly enriched for small RNAs. The genome mapping file for the *E. histolytica* HM-1:IMSS small RNA dataset was used to generate the mapping counts for each supercontig in R using base functions. The supercontig number, size and number of mapped small RNAs are listed. **Table S2.** Analysis of paired or clustered protein coding genes that have small RNAs mapping to them. Listed features include gene name, sRNA orientation, contig number, whether genes are paired or clustered, genomic duplication segments, proximity to repeat regions, orientation (divergent/convergent/tandem), gene length, intergenic distance between paired/clustered genes, number of sRNAs mapped to each gene, small RNA density on the gene, number of sRNAs mapped to the intergenic region, and the sRNA density on the intergenic region. **Table S3.** Small RNA reads map to exon-exon junction and intron from HM-1:IMSS EhAGO2-2 IP small RNA library. Listed are numbers of unique small RNA reads in each category. **Table S4.** Small RNA density on EHI_135940 and EHI_197360. Listed are the number of small RNA mapped to exons and introns and the calculated small RNA density on these regions for each gene. **Table S5.** A global assessment of small RNA regulated genes in *E. histolytica*. Genes to which small RNAs mapped in either the antisense or sense and antisense orientation were analyzed for their expression data using previously published microarray data. The number of genes on the Affymetrix microarray and those with normalized array data <0.2 (not expressed) under wild type conditions for *E. histolytica* HM-1:IMSS are listed. The number of genes not expressed under all conditions tested, those expressed in other *E. histolytica* strains (200:NIH and Rahman), those expressed under specific culture conditions (under different drug treatment and serum starvation) and those expressed in developmental stages are listed. Microarray data are adapted from [41-45]. **Table S6.** Oligonucleotide probes used for Northern blot analysis. The probe name, targeting gene/LINEs, orientation/position of the probe, and sequence of the probe are shown. S: sense; AS: antisense. **Table S7.** Primers used for strand specific RT-PCR analysis. Primer sequences, the tested genes, and orientation (F/R) are listed. Click here for file

Additional file 3: Table S8 Small RNAs mapping to protein coding genes containing introns or exon-exon junctions. We downloaded from AmoebaDB both genomic and mRNA gene sequences for all *E. histolytica* genes with at least one predicted intron. Small RNAs mapping to introns are reads that map to the genomic sequence but not the mRNA sequence. Small RNAs mapping to exon-exon junctions are reads that map to mRNA sequence but not the genomic sequence. Protein coding genes are shown in three categories: genes with only antisense small RNAs; genes with both antisense and sense small RNAs; genes with only sense small RNAs. The number of small RNAs mapping to a gene, intron, or exon-exon junction is indicated. Whether the intron has an in-frame stop codon or frame disruption is listed (Yes/NO). HM-1:IMSS EhAGO2-2 IP small RNA library dataset was used for the analysis. Only genes with ≥50 small RNAs are listed. Highly identical genes are indicated with same letter in column Paralog group. Click here for file
