# Supplementary material for: Small RNA pyrosequencing in the protozoan parasite Entamoeba histolytica reveals strain-specific small RNAs that target virulence genes
Source: BMC Genomics. 2013 Jan 25;14:53. doi: 10.1186/1471-2164-14-53 (PMC3610107; doi:10.1186/1471-2164-14-53)
Supplement: Additional file 1: Figure S1 — Flow-chart for small RNA sequence analysis. The pipeline for processing of the small RNA sequences is listed. Figure S2. The number of loci to which each small RNA maps. The genome mapping file for the E. histolytica HM-1:IMSS small RNA dataset was used to generate the mapping counts for each small RNA in R using base functions. The number of small RNA reads (y-axis) is plotted against counts of their mapped loci (x-axis). Figure S3. Nucleotide frequency at each position for the 17nt, 26nt and 28nt small RNA sequences. A 5′-G sequence predominance is evident for the aligned 26nt and 28nt reads but not for 17nt reads when the nucleotide frequency at each position is plotted. Figure S4. Representative supercontig view of the mapped small RNAs. Small RNAs were binned into windows of 500 bp along the supercontig. The counts of small RNA reads (y-axis) were plotted against a normalized supercontig length of one (x-axis). Three major patterns were seen for the graphs of the binned distributions. (A) Abundant small RNAs from clusters with several hot areas; these are mostly for the 19 supercontigs with ≥5000 small RNAs. (B) Small RNAs largely confined to isolated peaks in supercontigs. (C) Very low numbers of small RNAs in a given supercontig. Figure S5. Expression of protein coding genes with mapped small RNAs, using different cutoffs (no cutoff, ≥10, ≥25 and ≥50 small RNAs mapping to the gene). We plotted the microarray expression value for three sets of protein coding genes: those with only antisense small RNAs (AS only); those with both antisense and sense small RNAs (AS + S); those with only sense small RNAs (S only). Using both the ≥25 and ≥50 small RNA cutoffs, we observed significantly lower expression values among genes with AS or AS + S small RNAs. The number of genes for each category are listed. Figure S6. The density of small RNAs on paired or clustered genes and associated intergenic regions. Box-and-whisker plots showing small RNA density (small RNA/bp) on p [file 1471-2164-14-53-S1.pptx]

## Slide 1
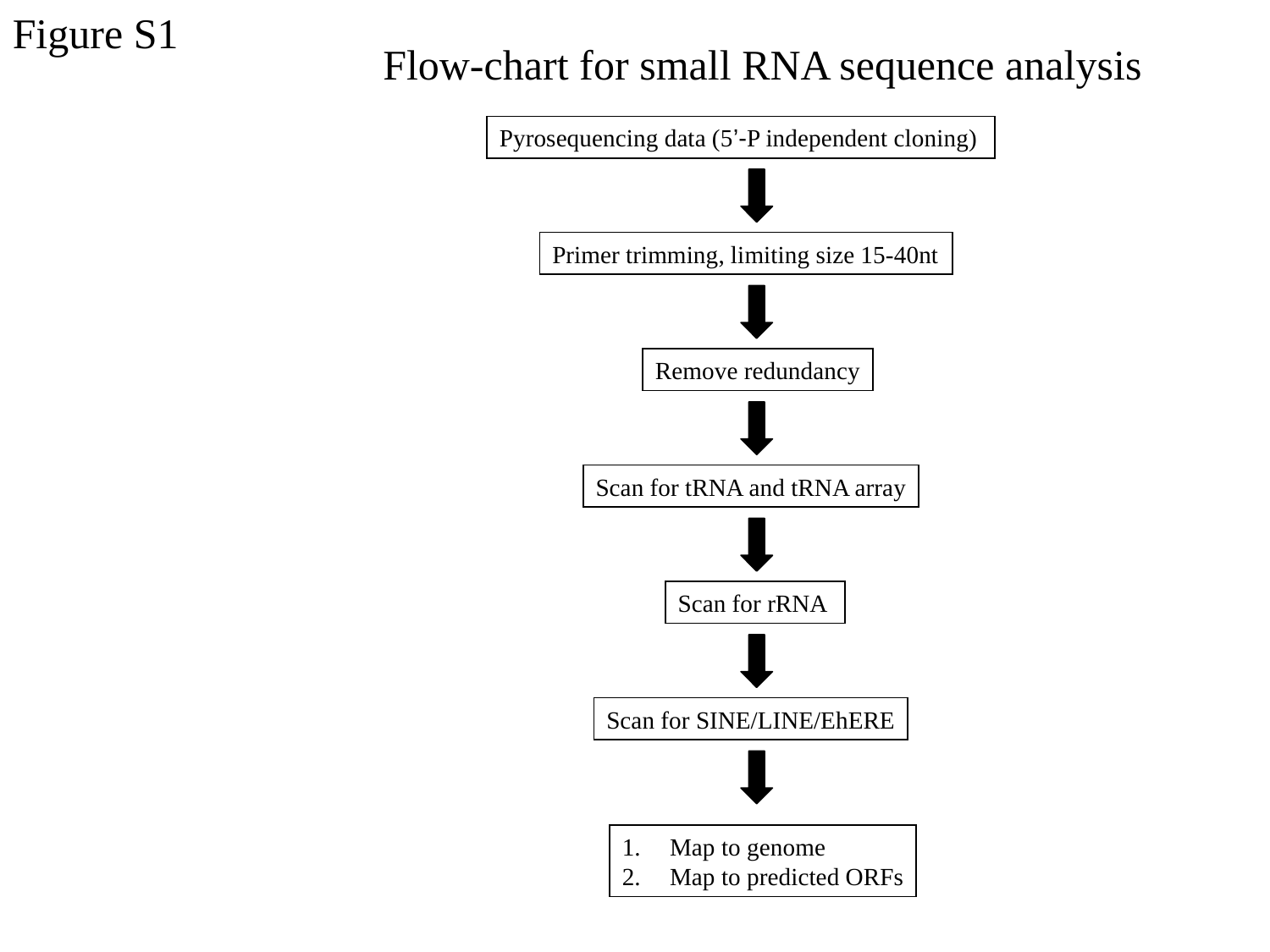

Figure S1
Flow-chart for small RNA sequence analysis
Pyrosequencing data (5’-P independent cloning)
Primer trimming, limiting size 15-40nt
Remove redundancy
Scan for tRNA and tRNA array
Scan for rRNA
Scan for SINE/LINE/EhERE
Map to genome
Map to predicted ORFs

## Slide 2
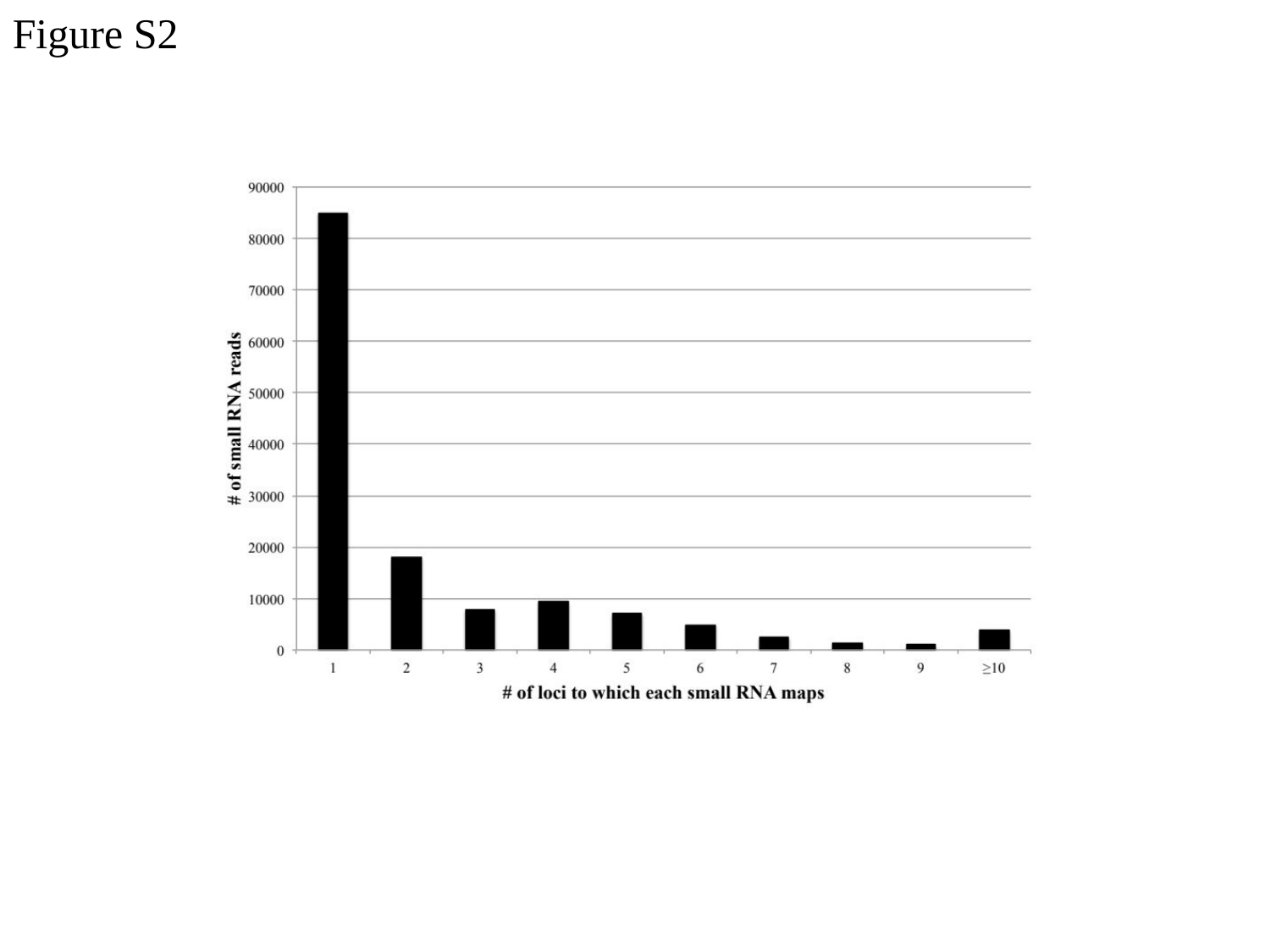

Figure S2

## Slide 3
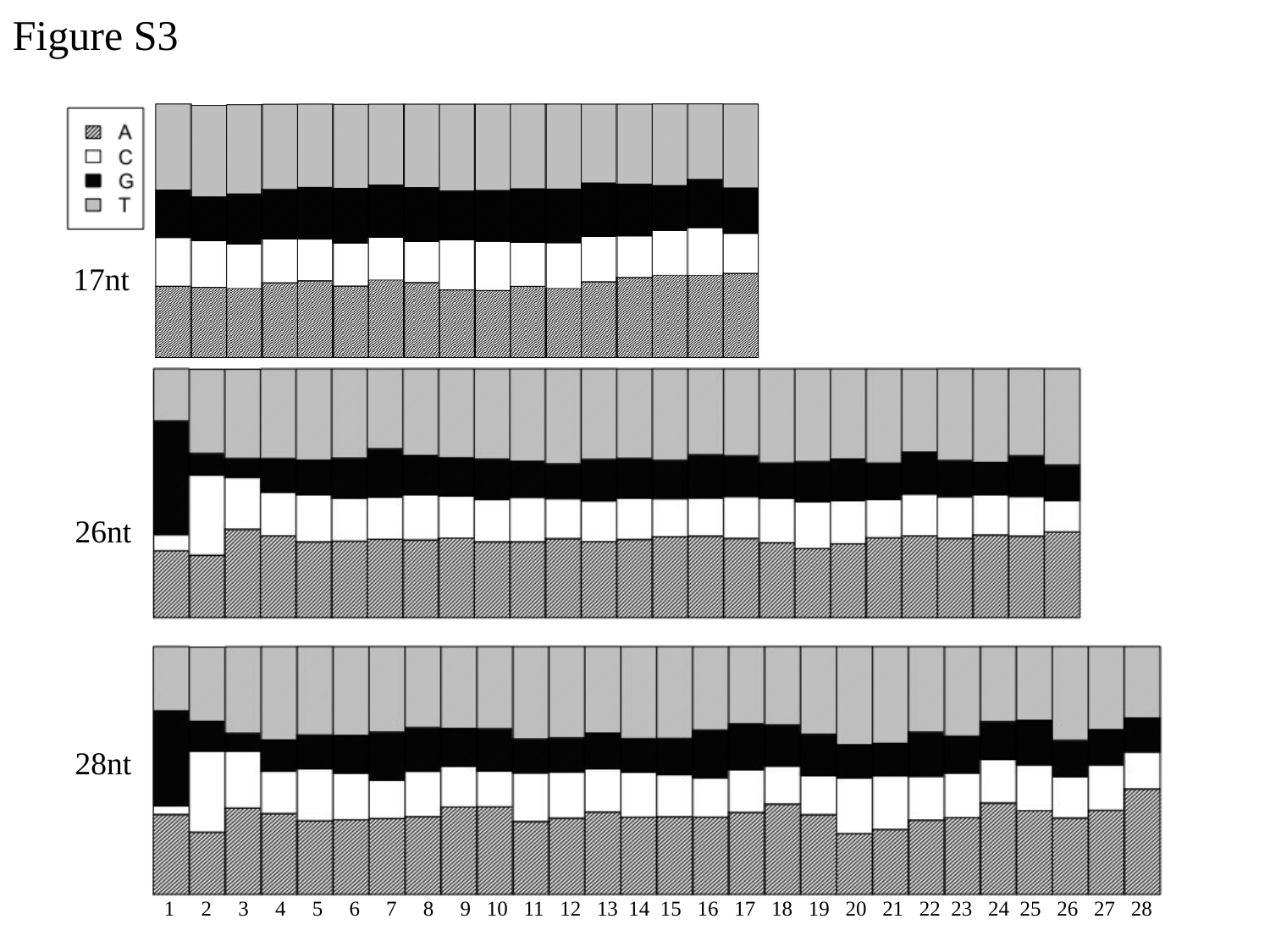

Figure S3
17nt
26nt
28nt
1 2 3 4 5 6 7 8 9 10 11 12 13 14 15 16 17 18 19 20 21 22 23 24 25 26 27 28

## Slide 4
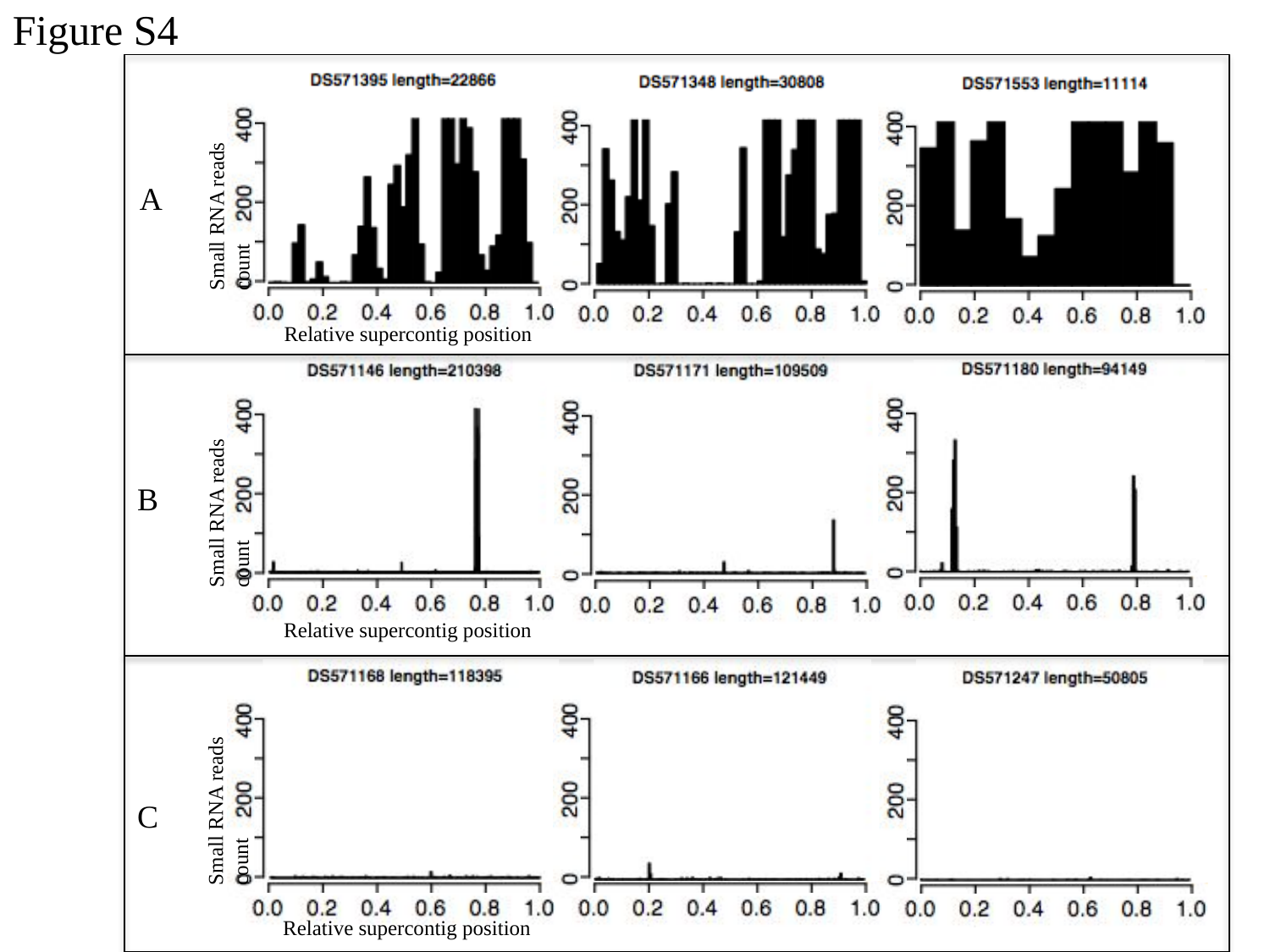

Figure S4
Small RNA reads count
A
Relative supercontig position
Small RNA reads count
B
Relative supercontig position
Small RNA reads count
C
Relative supercontig position

## Slide 5
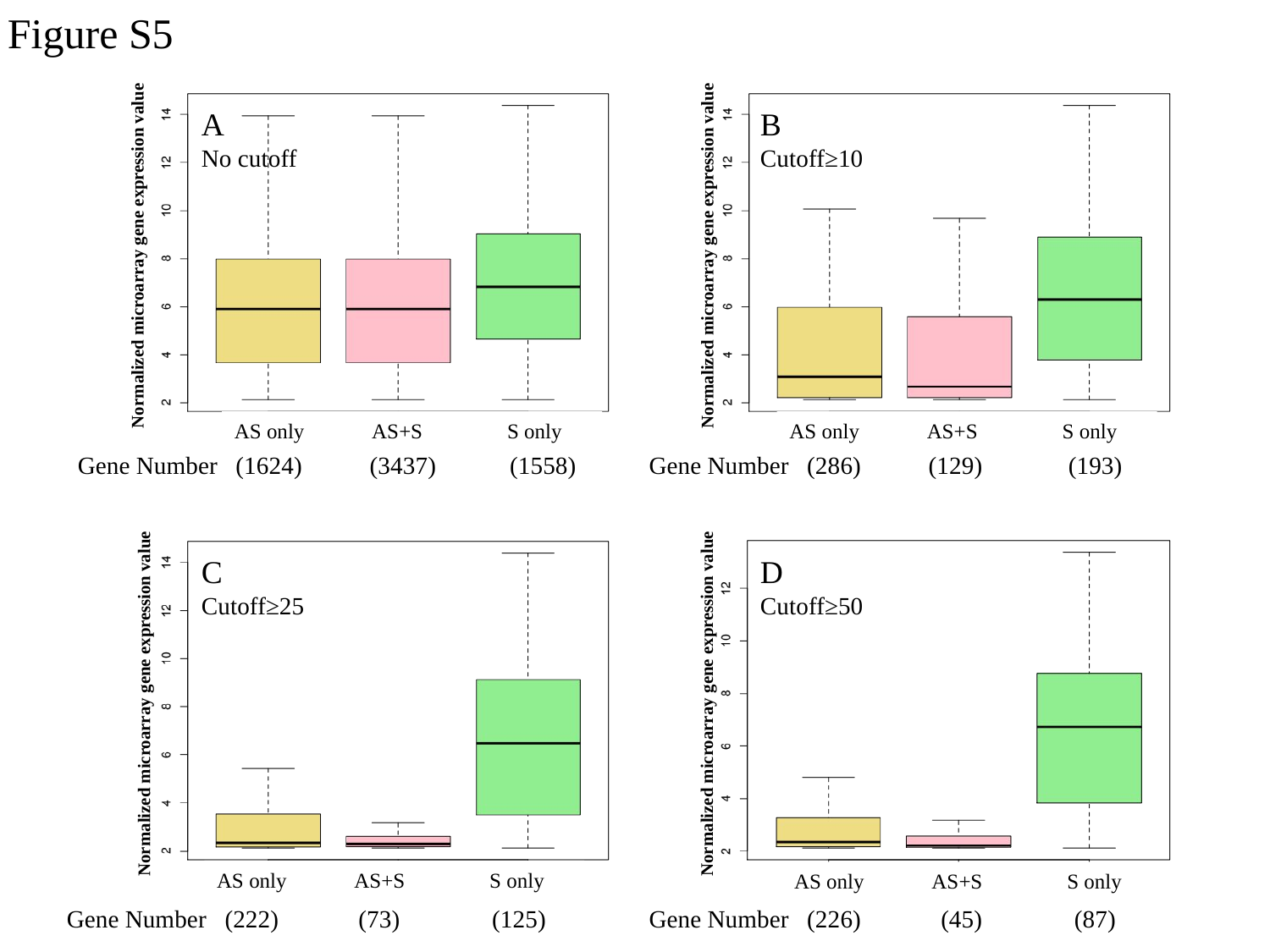

Figure S5
A
No cutoff
B
Cutoff≥10
Normalized microarray gene expression value
Normalized microarray gene expression value
AS only AS+S S only
AS only AS+S S only
Gene Number (1624) (3437) (1558)
Gene Number (286) (129) (193)
C
Cutoff≥25
D
Cutoff≥50
Normalized microarray gene expression value
Normalized microarray gene expression value
AS only AS+S S only
AS only AS+S S only
Gene Number (222) (73) (125)
Gene Number (226) (45) (87)

## Slide 6
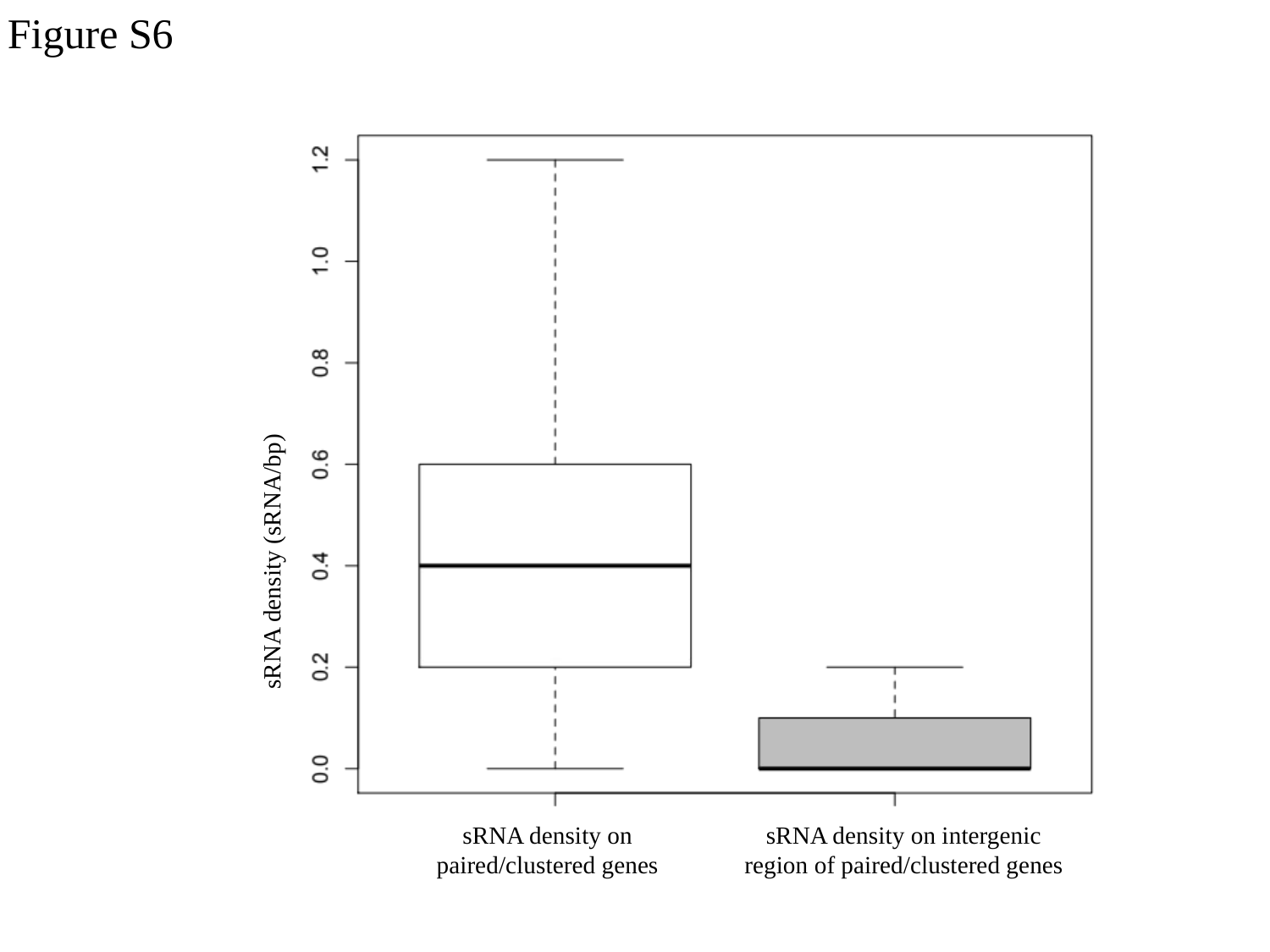

Figure S6
sRNA density (sRNA/bp)
sRNA density on paired/clustered genes
sRNA density on intergenic region of paired/clustered genes

## Slide 7
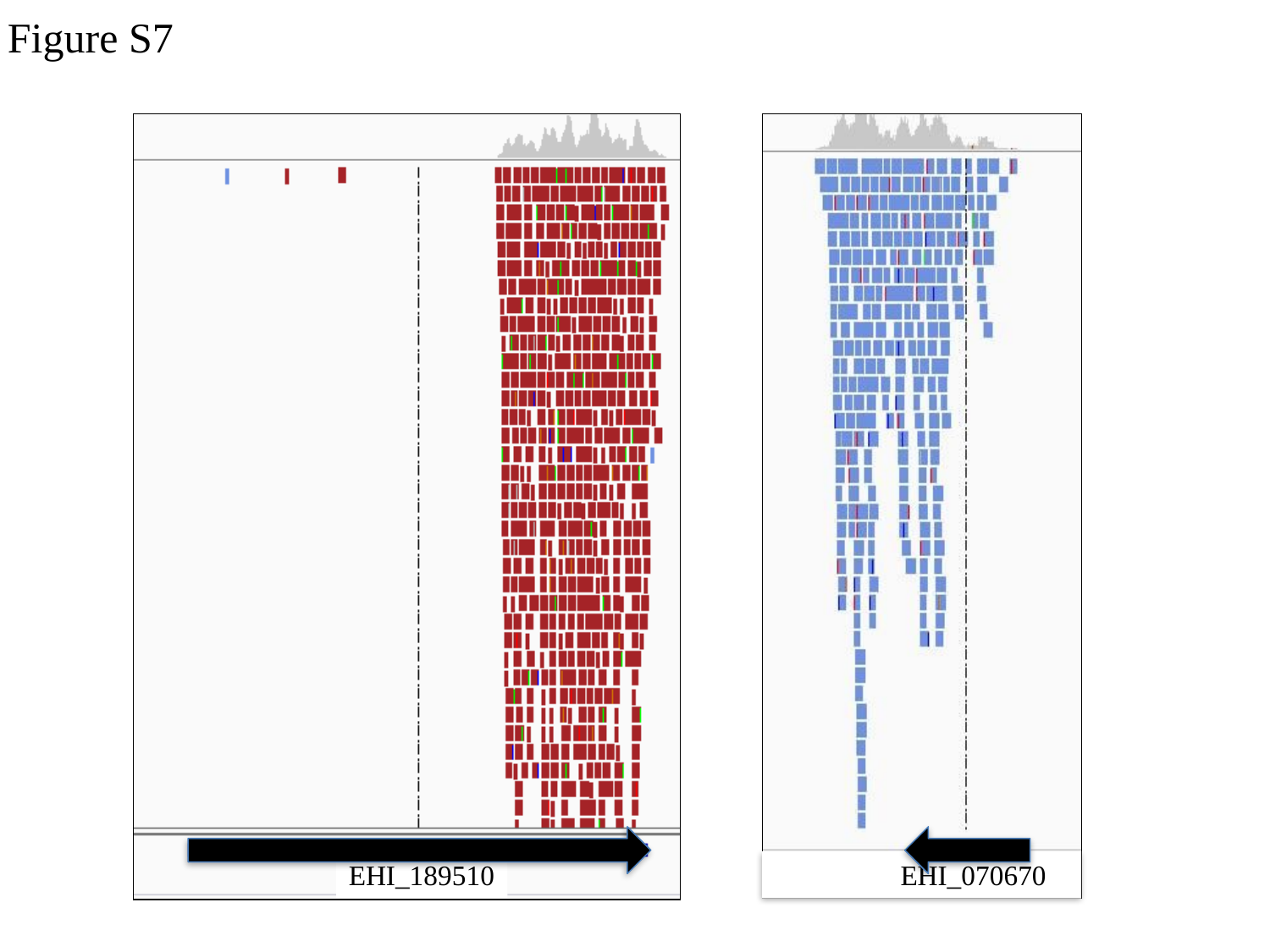

Figure S7
EHI_070670
EHI_189510

## Slide 8
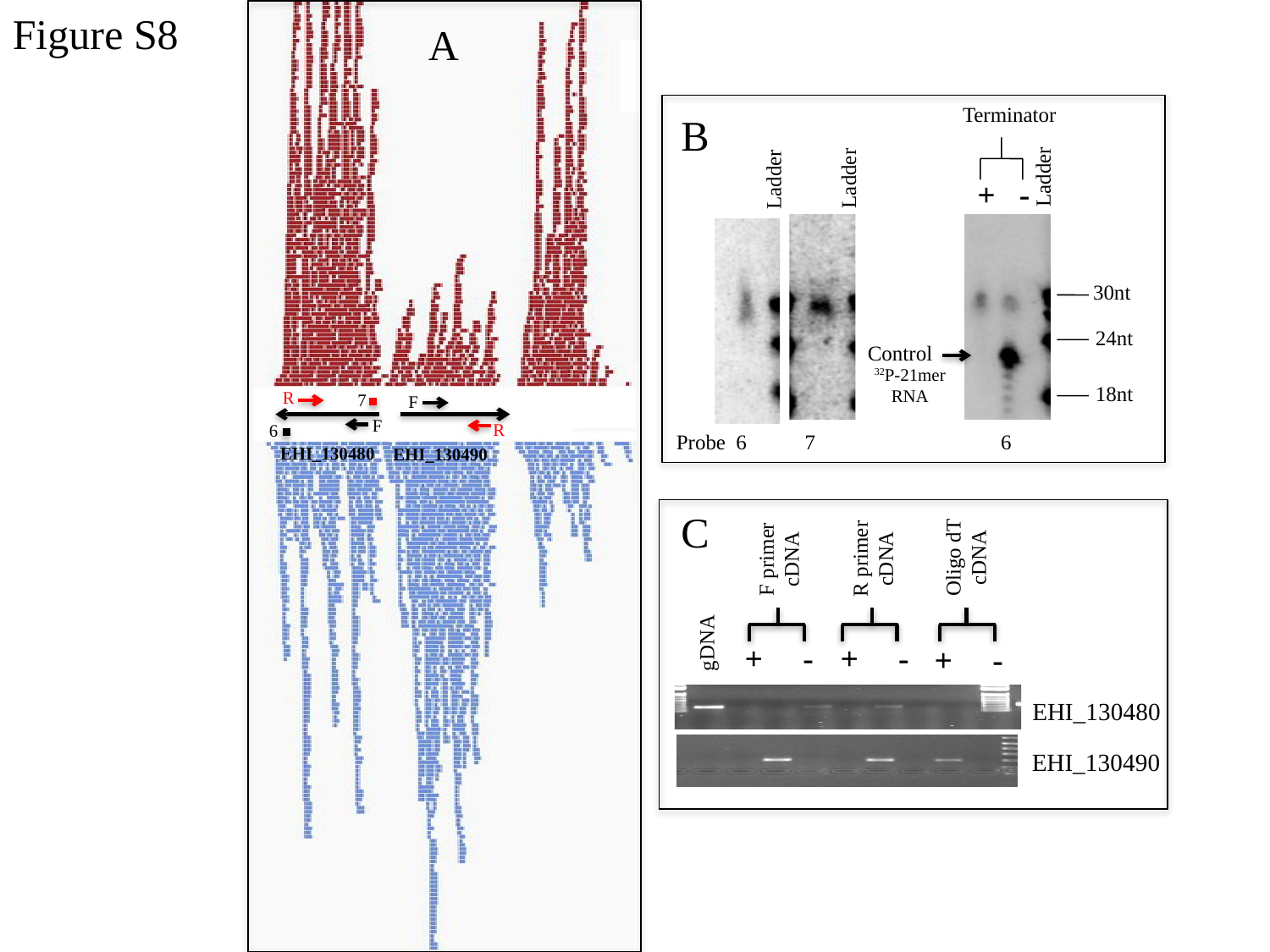

Figure S8
A
Terminator
B
Ladder
Ladder
Ladder
 + -
30nt
24nt
Control
32P-21mer RNA
18nt
R
7
F
F
R
6
 6
Probe 6 7
EHI_130480
EHI_130490
C
Oligo dT cDNA
R primer cDNA
F primer cDNA
gDNA
+ -
+ -
+ -
EHI_130480
EHI_130490

## Slide 9
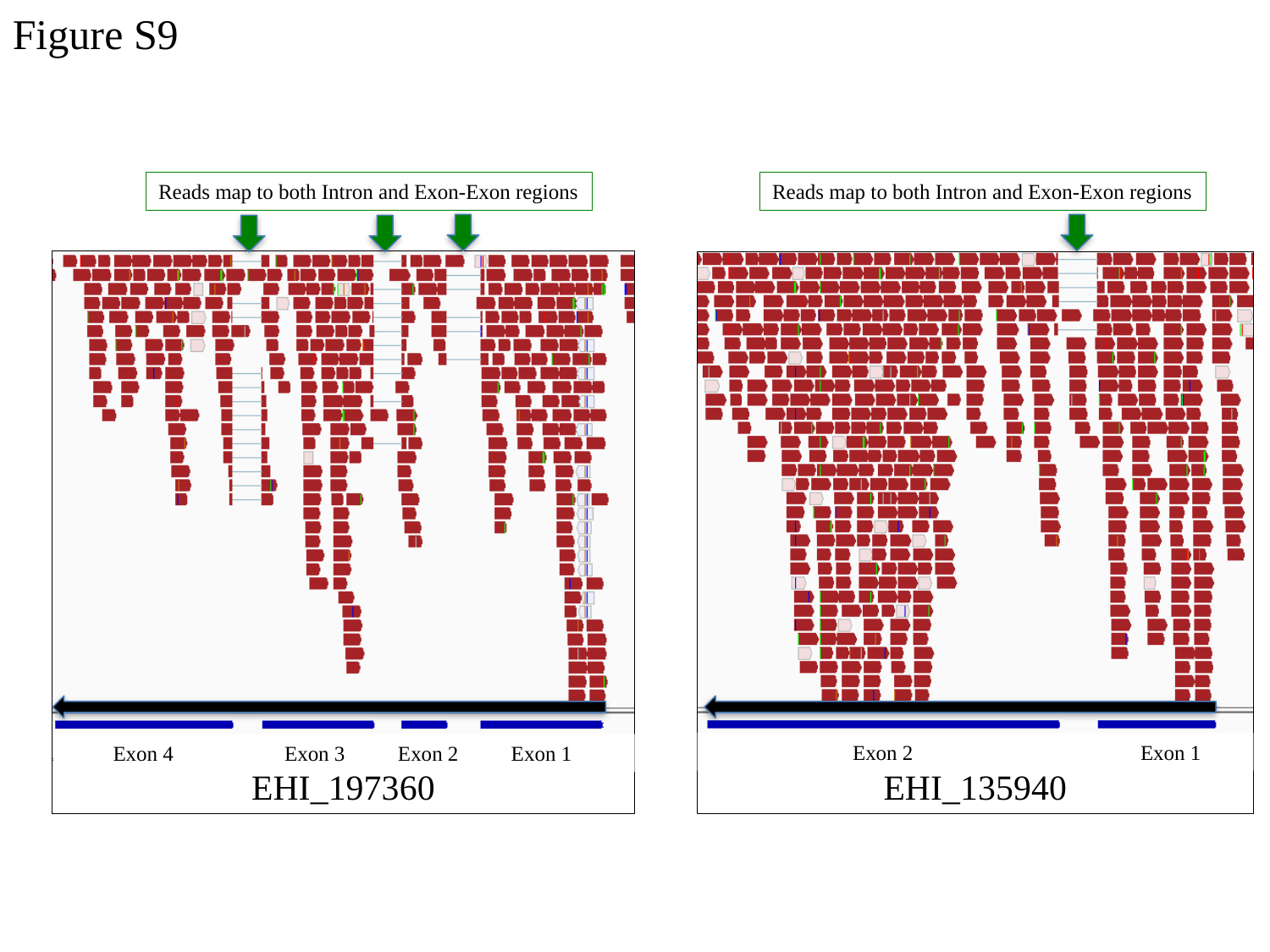

Figure S9
Reads map to both Intron and Exon-Exon regions
Reads map to both Intron and Exon-Exon regions
 Exon 2 Exon 1
 Exon 4 Exon 3 Exon 2 Exon 1
EHI_197360
EHI_135940

## Slide 10
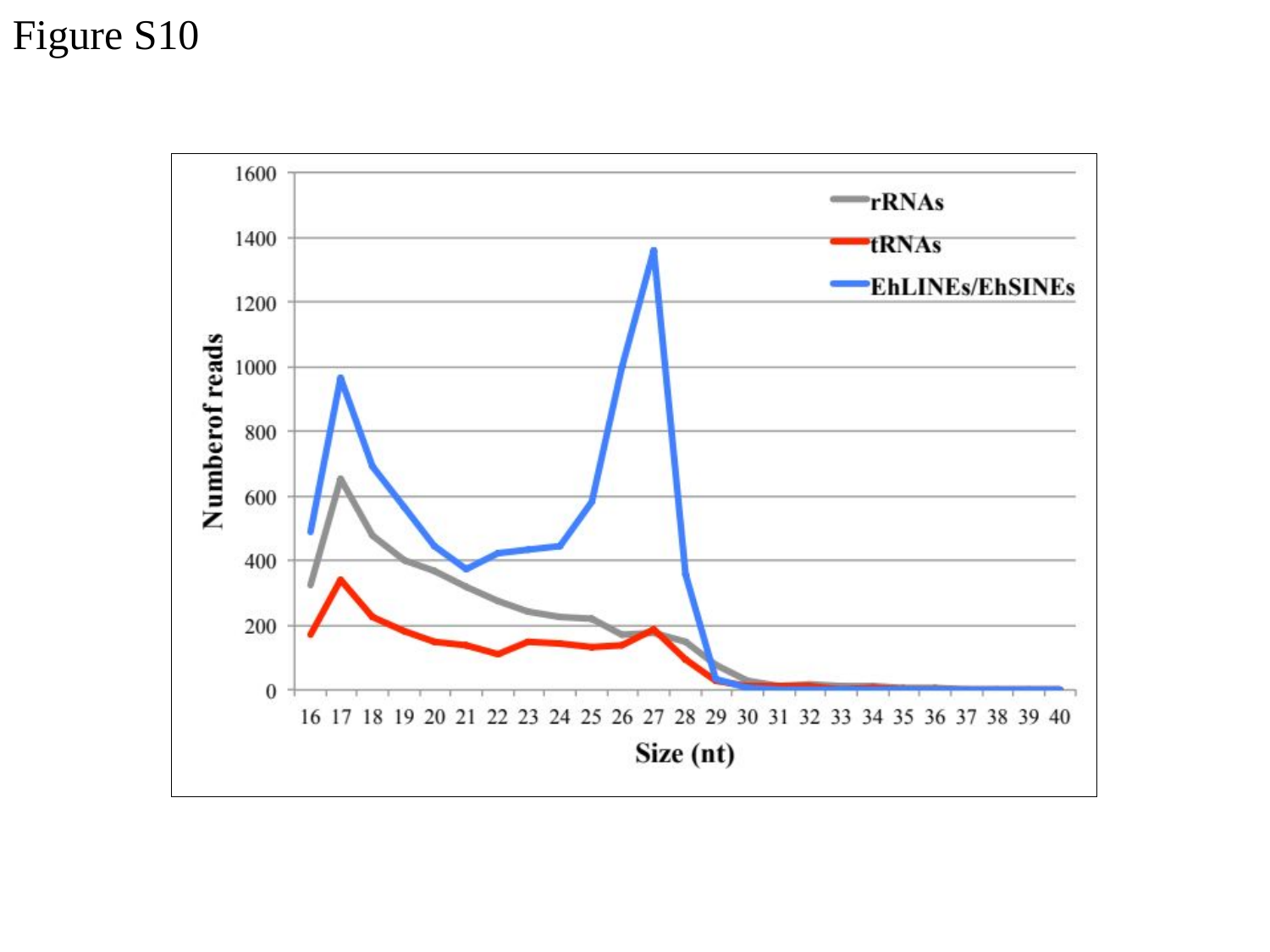

Figure S10

## Slide 11
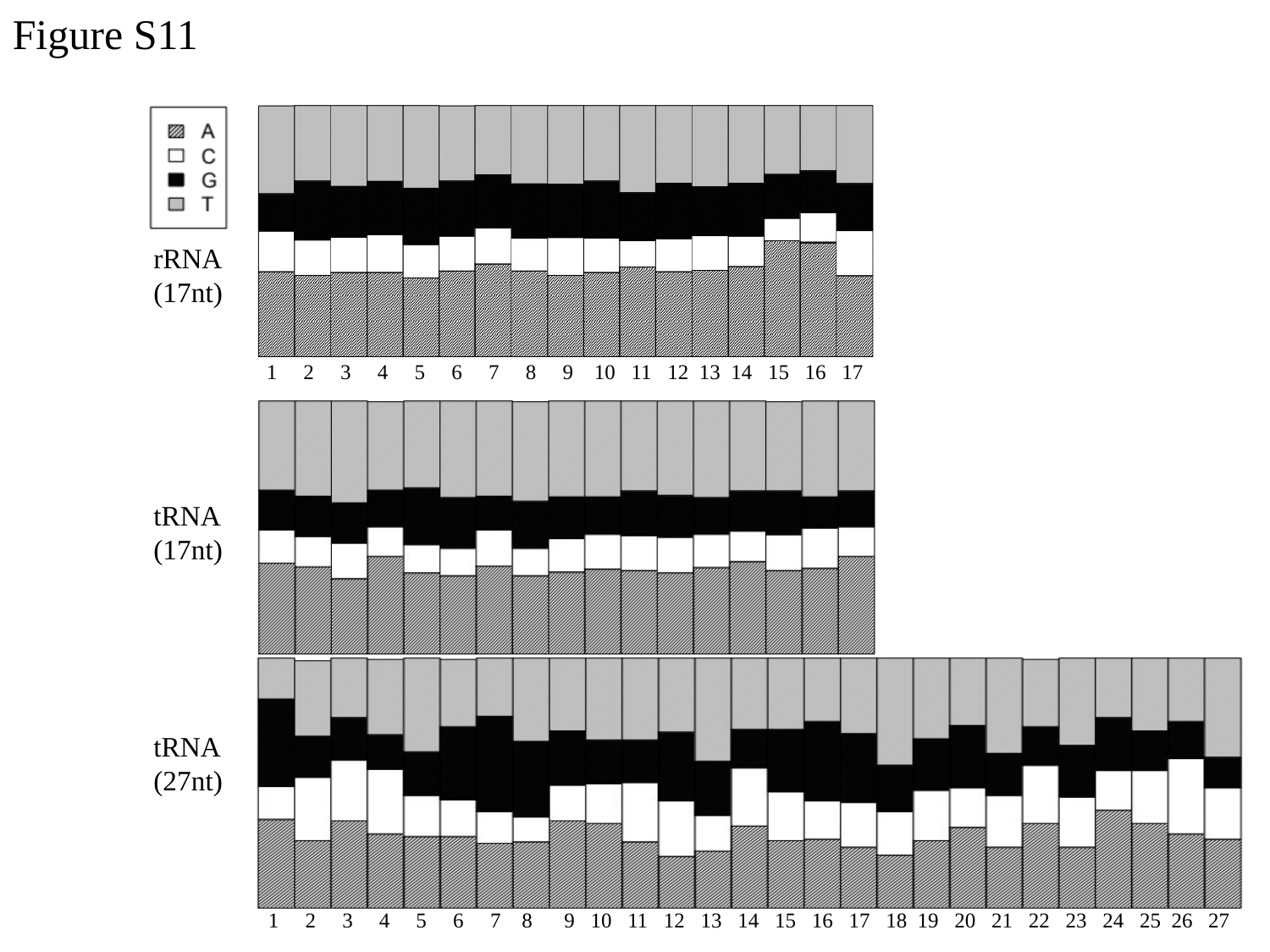

Figure S11
rRNA
(17nt)
1 2 3 4 5 6 7 8 9 10 11 12 13 14 15 16 17
tRNA
(17nt)
tRNA
(27nt)
1 2 3 4 5 6 7 8 9 10 11 12 13 14 15 16 17 18 19 20 21 22 23 24 25 26 27

## Slide 12
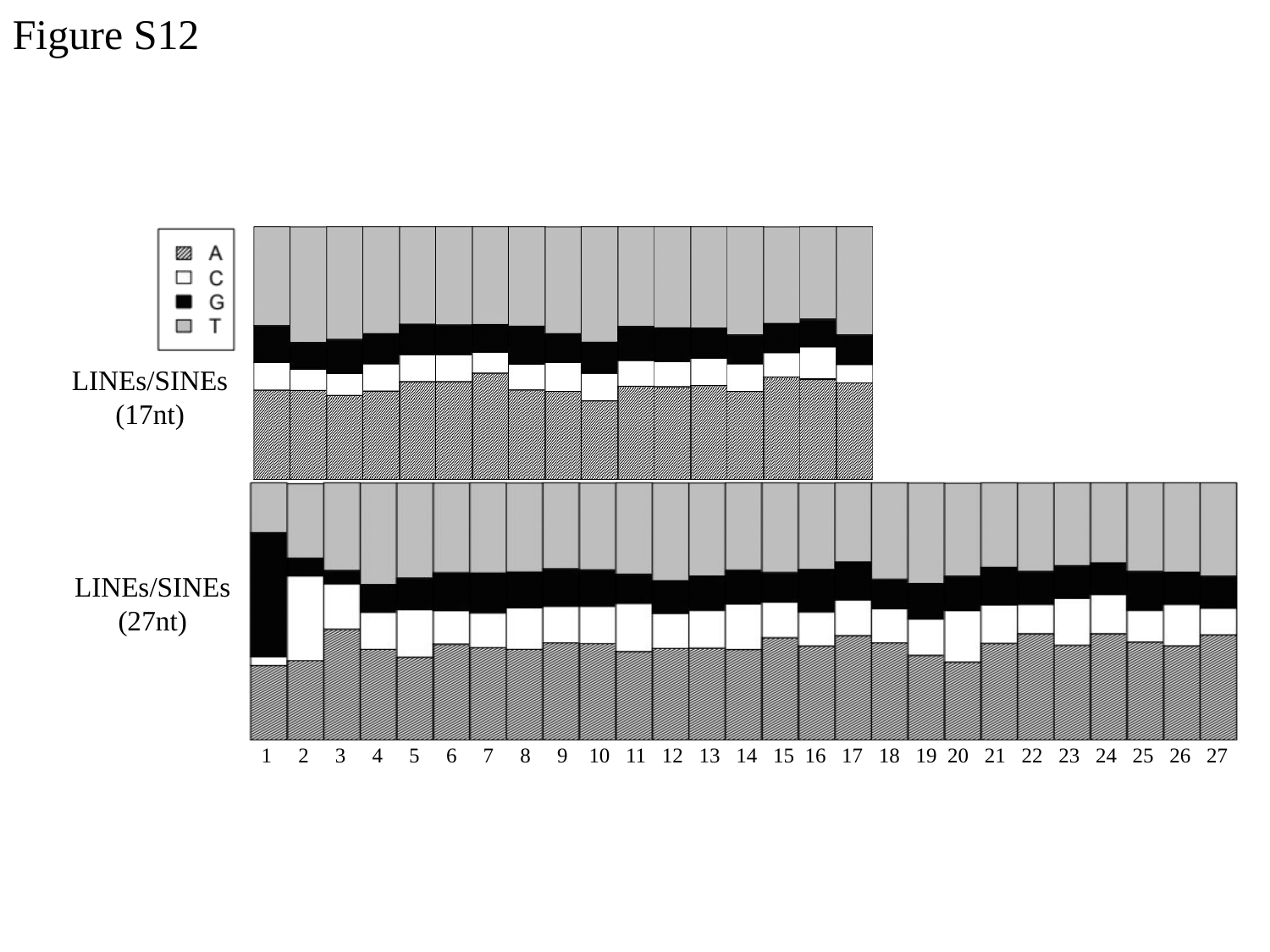

Figure S12
LINEs/SINEs
(17nt)
LINEs/SINEs
(27nt)
1 2 3 4 5 6 7 8 9 10 11 12 13 14 15 16 17 18 19 20 21 22 23 24 25 26 27

## Slide 13
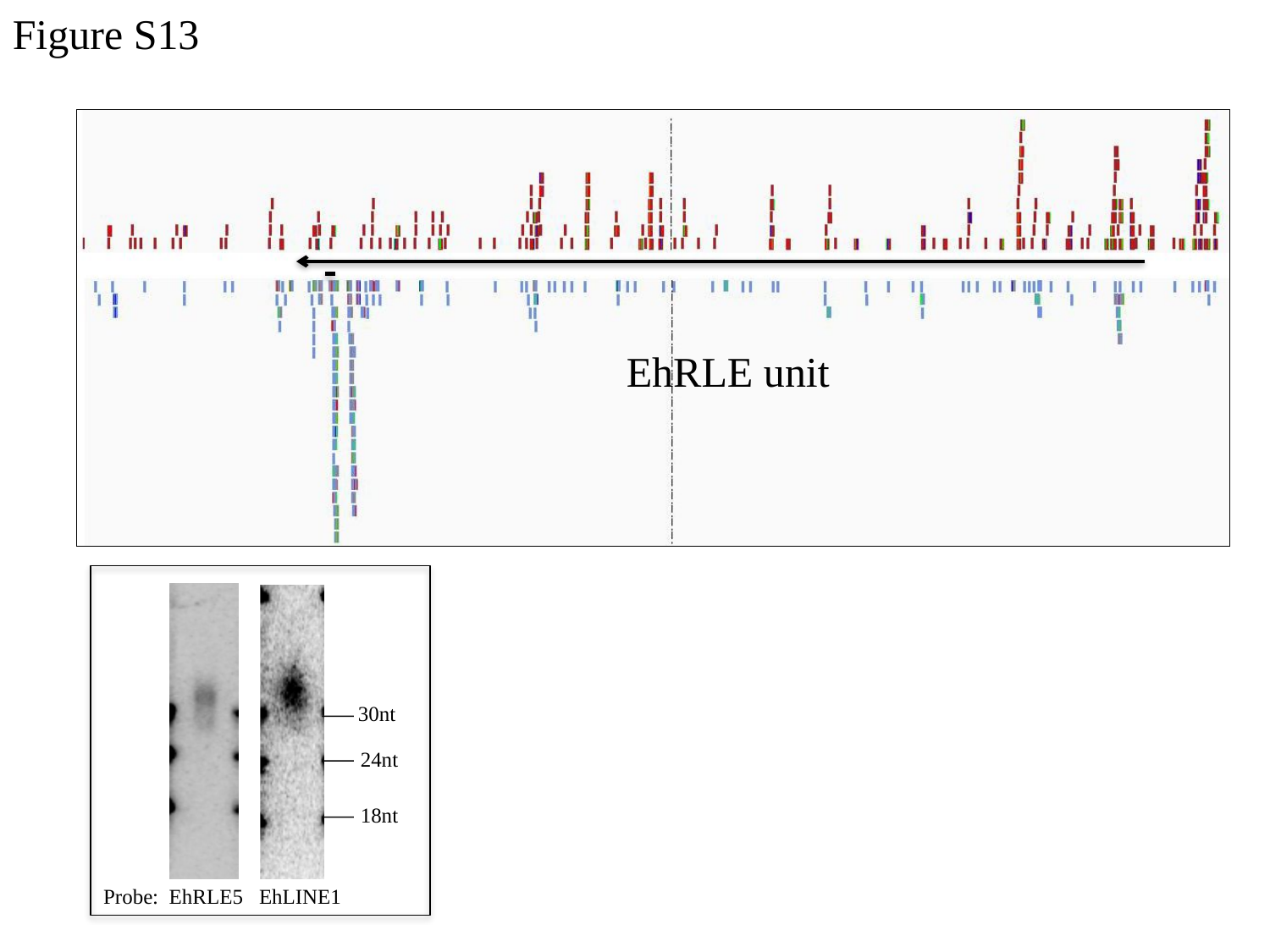

Figure S13
EhRLE unit
30nt
24nt
18nt
Probe: EhRLE5 EhLINE1

## Slide 14
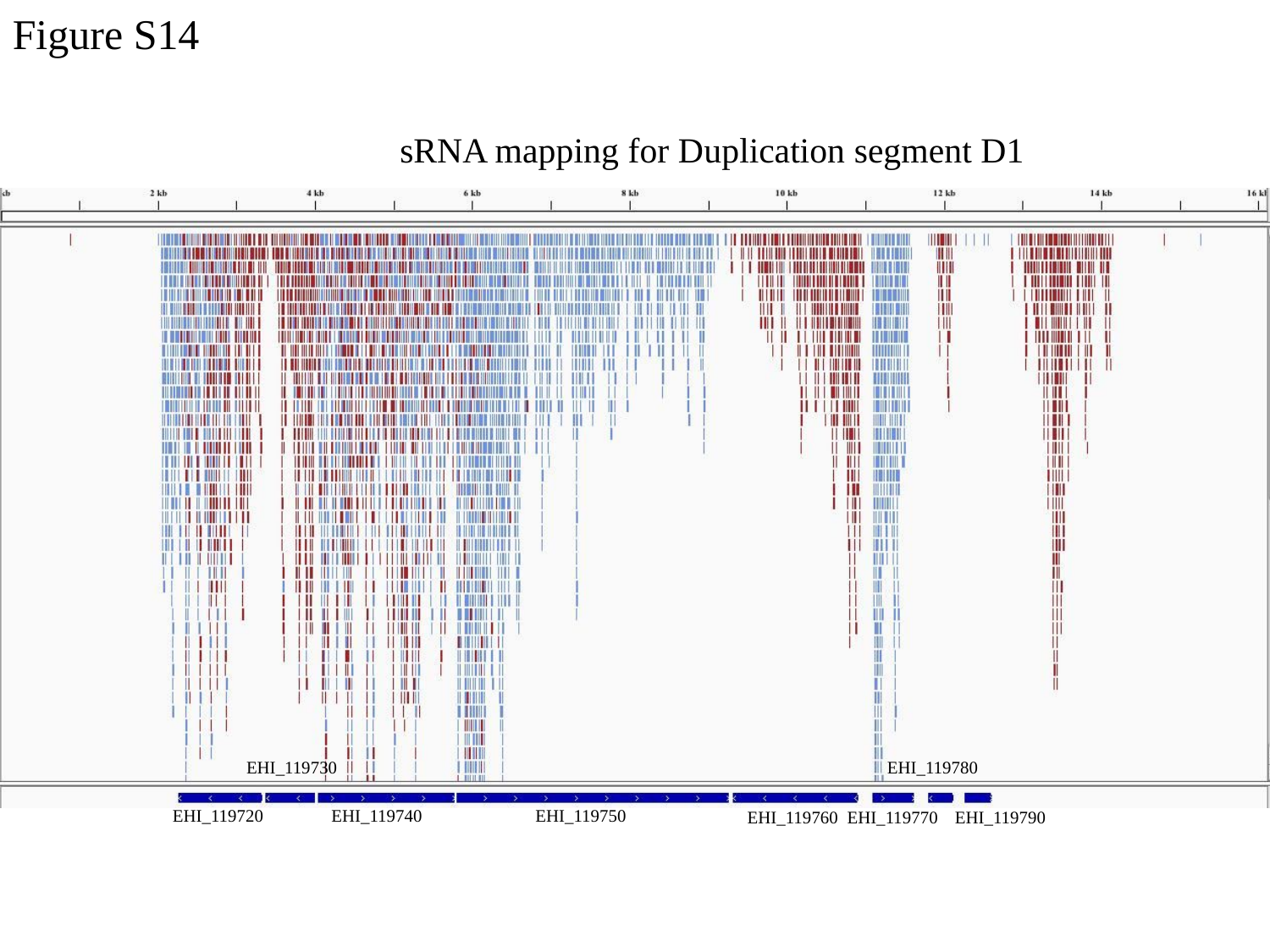

Figure S14
sRNA mapping for Duplication segment D1
EHI_119780
EHI_119730
EHI_119720
EHI_119740
EHI_119750
EHI_119760
EHI_119770
EHI_119790
